# Blood biomarkers to predict the onset of pre-eclampsia: A systematic review and meta-analysis

**DOI:** 10.1016/j.heliyon.2022.e11226

**Published:** 2022-11-04

**Authors:** Marianna Danielli, Roisin C. Thomas, Clare L. Gillies, Jiamiao Hu, Kamlesh Khunti, Bee Kang Tan

**Affiliations:** a Cardiovascular Sciences, University of Leicester, Leicester, LE1 7RH, United Kingdom; b Diabetes Research Centre, Leicester General Hospital, Leicester, LE5 4PW, United Kingdom; c Engineering Research Centre of Fujian-Taiwan Special Marine Food Processing and Nutrition, Ministry of Education, Fuzhou, Fujian, China; d NIHR Applied Research Collaboration – East Midlands (ARC-EM), Leicester General Hospital, Leicester, LE5 4PW, United Kingdom

**Keywords:** Biomarkers, Blood, Pregnancy, Pre-eclampsia, Early prediction

## Abstract

Pre-eclampsia is one of the most common pregnancy complications, and a major cause of fetal and maternal morbidity and mortality globally. Diagnosis currently takes place in the third trimester based on clinical symptoms. This systematic review and meta-analysis sought to determine the blood biomarkers that are associated with pre-eclampsia, and in particular, the biomarkers that could predict pre-eclampsia in early pregnancy. We searched the electronic databases (Medline, Embase, Cochrane Library) from inception up to March 2022. Prospective studies with 1000 or more participants that measured blood biomarkers to predict or diagnose pre-eclampsia have been included in this systematic review. Biomarkers’ measurements were considered from the first up to the third trimester, but not during labor. Data concerning pre-eclampsia, biomarker measurements and study characteristics were extracted. Meta-analysis was performed when possible. We found a total of 43 studies (assessing 62 different biomarkers in 18,170 pregnancies, have been included in this systematic review, and a total of 6 studies (assessing 2 biomarkers have been included in the meta-analysis). Statistical analysis was performed for PlGF and sFlt-1. Mean difference in PlGF levels between pre-eclampsia and healthy pregnancies, appear to increase as the pregnancy progresses. Results of sFlt-1 meta-analysis were inconclusive. No significant publication bias was identified. This is the most comprehensive and up to date systematic review and meta-analysis on this important topic on blood biomarkers for the early prediction of pre-eclampsia. Further This research highlights the urgent needed for further discovery research to identify blood biomarkers that could predict the development of pre-eclampsia.

## 1 Introduction

With a global incidence among pregnancies of 5%, pre-eclampsia (PE) is one of the major causes of maternal morbidity and mortality worldwide [1, 2]. As established by the International Society for the Study of Hypertension in Pregnancy (ISSHP), a diagnosis of pre-eclampsia can be made where there is new-onset hypertension (systolic blood pressure ≥140 mmHg or diastolic blood pressure ≥90 mmHg on two different occasions, 4 h apart) after 20 weeks of gestation with simultaneous proteinuria (≥300 mg/24 h urine collection or protein/creatinine ratio ≥30 mmol/L or dipstick urine reading ≥1) [3,4]. Severe clinical features of end-organ damage typical of pre-eclampsia include thrombocytopenia, hepatic and renal insufficiencies, pulmonary oedema, new onset of cerebral and visual symptoms and utero-placental dysfunction (such as growth restricted fetus) [3]. Eclampsia is a life-threatening complication, requiring urgent medical treatment, and is defined by the occurrence of generalized seizures in a woman with pre-eclampsia [5, 6]. Depending on the clinical onset of symptoms, pre-eclampsia can be classified as early-onset (first clinical presentation before 34 gestational weeks) and late-onset (disease develops at or after 34 weeks of gestation) [7]. More commonly, the former is associated with severe disease in both the mother and the child, including frequent development of fetal growth restriction [8, 9]; the latter is often accompanied by mild symptoms only [10].

As established by the Food and Drug Administration (FDA) and the National Institute of Health (NIH), a biomarker is ‘a defined characteristic that is measured as an indicator of normal processes, pathogenic processes or responses to an exposure or intervention’ [11]. Blood, urine and imaging biomarkers have been widely used during pregnancy, with maternal serum human chorionic gonadotropin (hCG) and progesterone being the most frequently adopted biomarkers to predict adverse pregnancy outcomes in early pregnancy [12]. The identification of a reliable, inexpensive, and non-invasive biomarker to predict the onset of pre-eclampsia in early pregnancy would be widely beneficial in reducing maternal and fetal complications by allowing better categorization of the disease according to its severity, monitoring its progression closely and detecting the disease before the clinical onset of any symptoms [13]. There may also be economical benefits, since early detection of disease could mean a reductions in healthcare costs [14]. However, a biomarker with the characteristics has not been identified yet. Currently guidelines exist to stratify women at high risk of pre-eclampsia in the first trimester simply by using maternal characteristics and medical history, but the sensitivity for disease identification is very low [15]. A more reliable screening for early (first trimester) detection of pre-eclampsia has been developed, which looks at mean arterial BP, Doppler ultrasound, and Placental Growth Factor (PlGF) blood measurements but has not been implemented into routine clinical practice due to the associated costs [4]. Finally, the combination of PlGF and sFlt-1 during the third trimester (but before 37 gestational weeks) is used to rule out a diagnosis of pre-eclampsia when the disease is suspected, as recommended by NICE guidelines [16].

The purpose of this systematic review is to investigate which blood biomarkers have been used for the diagnosis or early detection of pre-eclampsia to date.

## 2 Methods

### 2.1 Data sources and search strategy

This systematic review with meta-analysis was registered on PROSPERO (registration number CRD42020200589) and follows the Preferred reporting Items for Systematic Reviews and Meta-Analyses (PRISMA) guidelines [17], as shown in the result section (PRISMA flow chart) and in the Supplementary Material (PRISMA Checklist). A search strategy was developed for Medline database, and then adapted for Embase and the Cochrane Library. All the databases were searched from their inception up to March 2022. The complete search strategy can be found in Supplementary Material. The search strategy included terms related to “hypertensive disorders of pregnancy” combined with synonyms for “biomarkers”. The search was not limited by language. All the papers were imported into the Endnote reference manager tool, and duplicates were manually removed.

### 2.2 Eligibility criteria and study selection

All blood molecules measured during pregnancy were considered as a potential biomarker suitable for inclusion in this review including studies assessing gene expression or DNA/RNA analyses. The inclusion criteria were as follows: (1) prospective studies, presenting original data, (2) studies performed exclusively on pregnant women, (3) studies where the number of participants was greater or equal to 1000, (4) studies assessing blood (serum, plasma, or whole blood) biomarkers.

Case control and case series studies were excluded from the review. Studies assessing blood biomarkers only during/after labor were also excluded, since they are not relevant in terms of disease prediction. No language has been excluded a priori, and papers written in English, Italian, Spanish, French, Portuguese, and Chinese were evaluated by full text.

MD screened all the papers by title and abstract only, and retrieved papers considered as potentially relevant to this systematic review. Ful text of the identified studies were then evaluated and papers meeting the study selection criteria were classified according to the total number of participants. Only prospective original studies with more than 1000 participants were considered for the final analysis. The second reviewer (RT) independently checked the excluded and included papers. Any disagreement during the process was resolved through discussion and, when necessary, on the advice of a third author (CG).

### 2.3 Quality assessment and data extraction

The Newcastle-Ottawa Scale (NOS) for observational studies was used to assess the quality of the studies retrieved for the analysis (Supplementary Material) [18]. Publication bias was assessed using Begg’s and Egger’s tests and funnel plots [19, 20].

Data was independently extracted by two authors as per the Cochrane Handbook guidelines and reported following PRISMA guidance [21]. Data regarding study size, age, BMI, ethnicity, blood component used, gestational week (GW), and inclusion and exclusion criteria were extracted and imported into Excel spreadsheets. Pre-eclampsia was classified according to its onset (at either any gestational week or before 34 weeks of gestation) and, when reported, the values of each biomarkers measured have been included. Finally, a table for each biomarker was created. Where possible, values have been converted to the unit of measurement most frequently used among the selected studies.

### 2.4 Data synthesis and analysis

Random effect meta-analysis models were used to compare the predictive value of PlGF to diagnose pre-eclampsia in early, mid, and late pregnancy. Due to the way the data was presented in the included studies assessing PlGF, early pregnancy was arbitrarily defined as ≤ 18 gestational weeks, mid-pregnancy between 19 and 25, and late pregnancy when the measurements have been performed after the 26^th^ week of gestation.

Similarly, a random effect meta-analysis was performed to compare sFlt-1 values between early (<18 GW) and late/mid pregnancy (at or after 18 weeks’ gestation). Additional analysis was performed with the aim of showing sFlt-1 distribution over the first and second trimester (≤17 GW) among studies.

No further meta-analyses were performed for the remaining biomarkers identified due to a lack of data or inconsistent reporting across studies.

### 2.5 Included biomarkers

The main biomarkers object of this systematic review and meta-analysis include Placental Growth Factor (PlGF), Pregnancy-associated plasma protein 1 (PAPP-A), soluble Fms-Like Tyrosine Kinase-1 (sFlt-1), beta human chorionic gonadotropin (β-hCG), sFlt-1/PlGF ratio, leptin, soluble endoglin (sEng), alpha-fetoprotein (AFP), and Uric Acid (UA). An overview of their characteristics is presented in Table 1.

**Table 1:** Main biomarkers included in this review.

Biomarker	Characteristics	Role in pregnancy	Role in PE	Limitations in PE detection
PlGF	Vascular endothelial growth factor (VEGF) family member Expressed in the placental tissue [22] Mainly produced by trophoblasts [22]	Pivotal role in pregnancy angiogenesis [22] Serum levels increase at the end of the first trimester, peak at week 30, and decrease in the third trimester [23]	Used to rule out pre-eclampsia between 20 and 36 + 6 GW in women with symptoms but not meeting diagnostic criteria [24] Decreased serum levels between 28 and 30 GW [24] Lower levels in the first trimester [23]	Low sensitivity (32%) [23] High false-positive rate (5%) [23]
PAPP-A	Also known as pappalysin-1 High weight molecular glycoprotein (metalloproteinase) [25] Produced by placenta [25]	Levels rise as the gestation progresses [25] Routinely measured as part of the aneuploidy screening [26, 27, 28, 29] Lower levels associated with a higher chance of chromosomal abnormalities [26, 27, 28, 29]	Lower serum levels increase the risk of developing pre-eclampsia [30, 31, 32]	Low sensitivity [33] High false positive rate [33]
sFlt-1	Antiangiogenic tyrosine kinase protein [34] Binds PlGF and VEGF and blocks their pro-angiogenic effects [34] Mainly produced by placental tissue [35]	Higher serum levels in pregnancy (peak after 36 GW) [34]	Higher levels in PE for up to six months after delivery [34, 36, 37, 38, 39]	Used in combination with PlGF
β-hCG	Glycoproteic hormone [25] Produced by trophoblasts [25]	Promotes embryo implantation [25] Routinely used for early detection of pregnancy Levels peak at 8–9 GW, and decrease throughout pregnancy [40, 41] Used as part of the quad screening – levels higher in Trisomy 21, lower in Trisomy 13 and Trisomy 18 [42,43]	Higher levels in pre-eclamptic women as compared to healthy controls in the second trimester [44]	No definite link has been established [45, 46, 47, 48]
sFlt-1/PlGF			Diagnostic aid for pre-eclampsia detection [24] Higher levels in women who will develop pre-eclampsia [16, 49, 50]	Use regulated by NICE guidelines [24]
Leptin	Associated with adipogenesis and obesity [51, 52] The most abundant and studied adipokine in humans [51, 52]	Role has been investigated [53] Increased levels in pregnancy [54]	Higher levels in pre-eclampsia [54]	Studies on larger scale required
sEng	Transmembrane glycoprotein [55, 56] Actively involved in angiogenesis and inflammation [55, 56]	Role played in early pregnancy complications is an active area of research [57]	The higher the concentration, the more severe pre-eclampsia [58, 59, 60]	Studies on larger scale required
AFP	Albumin-like glycoprotein [61] Produced by the liver [61]	Levels measured as part of the triple and quadruple tests for chromosomal anomalies detection [62, 63]	No consistent correlation found [64, 65]	Studies on larger scale required
UA	Used to monitor a vast spectrum of systemic diseases	Concentrations decrease in first trimester and progressively increase up to delivery [66]	Evidence of hyperuricemia and pre-eclampsia [67]	Studies on larger scale required

## 3 Results

### 3.1 Study characteristics

As shown in the PRISMA flow chart of the literature (Table 2), an initial search strategy returned 15437 records in total. 3985 duplicates were removed manually after having sorted all papers by title. Of the 11452 papers evaluated by title and abstract, 8927 were excluded because they did not meet the selection criteria (animal studies, retrospective studies, reviews, letters, commentaries, surveys, editorials, abstracts, oral presentations, posters, systematic reviews, no biomarker measured, studies performed outside pregnancy, irrelevant topic). A total of 2545 full-text articles were assessed for eligibility, and among those 2501 were excluded because they were case control studies, case series studies, studies with a population size of less than 1000, studies not measuring biomarkers in the blood, or pre-eclampsia was not an outcome of interest. The full text of 8 papers were in Chinese, and they were assessed by one of the authors (JH). Overall, 43 studies met the criteria and were used for data extraction 46, 68, 69, 70, 71, 72, 73, 74, 75, 76, 77, 78, 79, 80, 81, 82, 83, 84, 85, 86, 87, 88, 89, 90, 91, 92, 93, 94, 95, 96, 97, 98, 99, 100, 101, 102, 103, 104, 105, 106, 107, 108, 109, 110, and six were suitable for meta-analysis [68, 72, 87, 96, 100, 108].

**Table 2:**
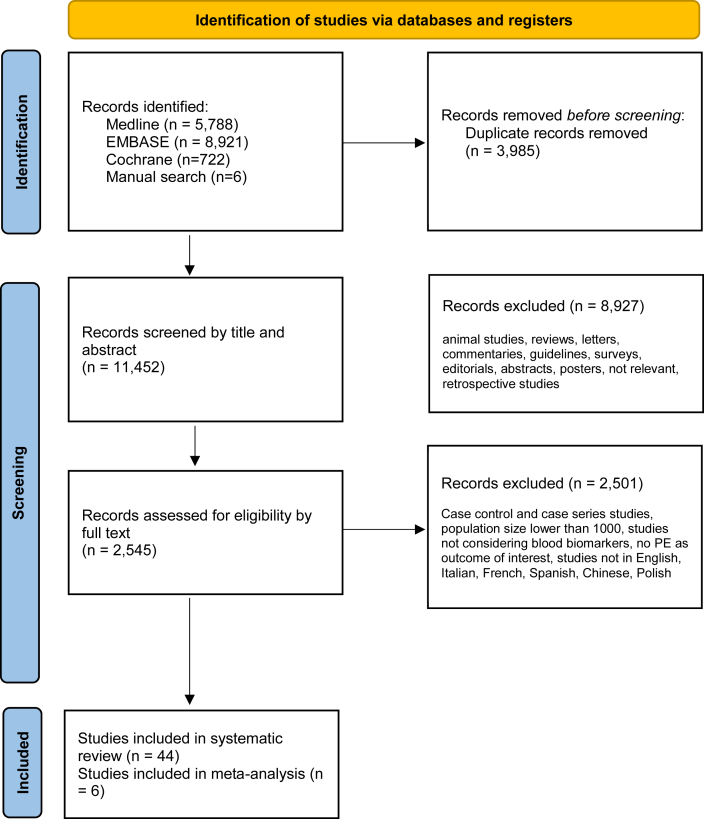
PRISMA flow chart.

A total of 18,170 pregnant women were considered for this systematic review, of whom 7295 had either pre-eclampsia or gestational hypertension. As shown in Supplementary Table S2, the ethnicity was mixed in most of the studies. Seven out of the 43 studies recruited women from more than one country and were thus classified as international. Of the remaining 31, 13 were based in Europe, 12 in Asia, seven in America, four in Australia, and one in Africa. Despite there being no limitation on the year of publication in the search strategy, most studies were conducted between 2002 and 2022, with only one taking place in 1993. Mean age ranged from 23.1 to 33.4 years, while BMI ranged from 20.5 to 27.8 kg/m^2^. Data regarding age and BMI were omitted by 3 and 10 studies respectively. Serum was the blood component analyzed most frequently, whilst plasma only was used in 6 studies. Both plasma and serum were analyzed in one study, and blood specifics were not mentioned in 4 studies. Immunoassay techniques were used by 29 studies to detect biomarkers, with the enzyme-linked immunosorbent assay (ELISA) being the most used (in 15 studies). Other methods included polymerase chain reaction, high performance liquid chromatography, calorimetric techniques, nephelometry, refractometry, and tandem mass spectrometry. The studies included in this systematic review mainly focused on biomarkers measured in early pregnancy, thus between the 1st (1–12 GW) and 2nd (13–27 GW) trimester of gestation; only 7 out of the 43 studies provided values from the 3rd trimester (28–40 GW). Most studies analyzed blood withdrawn during the first antenatal booking scan, performed between 10 and 14 weeks of gestation. Three studies analyzed samples exclusively during the 1st trimester. Except for 2 studies which included women at risk of developing PE [68, 101], all the studies included in this systematic review focused on healthy singleton pregnancies. one study did not provide inclusion nor exclusion criteria [89]. Data on study design were also extracted and included in the table of study characteristics (Table S2).

A total of 62 biomarkers were included in this review. As illustrated in Supplementary Table S4, most of the studies measured more than one biomarker with the most analysed was PlGF. PlGF measurements were an outcome of interest in 19 studies, followed by PAPP-A (12 studies), hCG (9 studies), and sFlt-1 (8 studies). Only 9 papers provided separate biomarker measurement data for early-onset pre-eclampsia. A total of 10 studies reported no numeric data for the biomarkers studied.

### 3.2 Placental Growth Factor (PlGF)

Nineteen studies measured PlGF as a biomarker in both healthy and pre-eclamptic women [68, 69, 71, 72, 77, 78, 81, 82, 87, 91, 92, 96, 99, 100, 103, 105, 107, 108, 110] (Table 3). A total of 83,047 women without pre-eclampsia and 3,756 with pre-eclampsia had their serum PlGF measured in the included studies. Of those, six papers reported data suitable to be analyzed [68, 72, 87, 96, 100, 108]. When provided, PlGF measurements were higher in healthy pregnancies as compared to pre-eclamptic ones, in all studies apart from Sonek et al. [82], in which the mean value of PlGF was 1.01 (0.81–1.27) MoM in the control group and of 1.07 (0.84–1.28) MoM and 0.68 (0.38–1.17) MoM in women developing late onset and early onset pre-eclampsia respectively. Three out of fifteen studies did not provide any numeric data for PlGF levels [69, 81, 103]. PlGF was measured at different timepoints, from the first up to the third trimester of pregnancy. Some studies provided measures of odds ratio (OR), positive and negative likelihood ratio (PLR, NLR), positive and negative predictive value (PPV, NPV), sensitivity and sensibility. When available, these data were extracted and included in Table 3.

**Table 3:** Placental growth factor (PlGF).

UNIT OF MEASUREMENT	STUDY ID	STUDY DESIGN	VALUE			OR, PLR, NLR, PPV, NPV	SENSITIVITY, SPECIFICITY	TRIMESTER (GW)	NUMBER OF PARTICIPANTS		
			no condition	PE					no PE	PE	
				any GW	⩽34 weeks (early onset PE)					any GW	⩽34 weeks (early onset PE)
pg/ml	Widmer et al. (2015)	Multicenter, prospective	84.6 (35.4–167.8)	55.9 (23.5–117.4)	59.9 (24.8–122.2)	PLR, NLR, ORs, sensitivity and specificity provided by cut-off only		1st–2nd (<20 GW)	4929	198	47
			456.3 (280.5–696.0)	253.7 (122.4–396.0)	153.6 (81.7–356.0)			2nd (23–27 GW)			
			513.8 (276.1–894.2)	152.5 (79.0–249.7)				3rd (32–35 GW)			
	Honigberg et al. (2016)	Multicenter, prospective,	14.6	11.8		PPV = 10.2% NPV = 93.6%	56.4%, 56.4%	1st-2nd (10–18 GW)	2153	184	18
			37.3	26.8		PPV = 11.9% NPV = 94.7%	60.9%, 61.0%	2nd (18–26 GW)			
			-6.3	-15.8, p < 0.0001		PPV = 10.4% NPV = 94.7%	59.1%, 59.1%	2nd-3rd (26–35 GW)			
	Coolman et al. (2012)	Monocenter, population-based prospective cohort	43.5 (21.9–120)	36.3 (17.4–111.6)		n/a		1st-2nd (<18 GW)	7327	167	23
			203 (111–415)	149 (64.8–341)				2nd (18–25 GW)			
	Herraiz et al. (2018)	Monocenter, prospective observational cohort	378.1 (median IQR 264.5)	96.2, median IQR 121.6 (intermediate), 262.5, median IQR 357.2 (late)	38.9, median IQR 41.2	n/a		2nd-3rd (24–28 GW)	5365	236	14
	Schneuer et al. (2013)	Monocenter, population-based prospective cohort	24.1 (18.3, 31.7)	20.7 (17.2, 32.6)		PPV = 3.7% (1.2, 8.4) NPV = 97.5% (96.8, 98.1) PLR = 1.47	7.4% (2.4, 17.3)	1st-2nd (12–14 GW)	2468	68	
	Boutin et al., 2020	Monocenter, prospective cohort	35.5 (27.3–46.3)		21.6 (15.0–31.0)	n/a		1st (11–13 GW)	3764	225	29
	Kusanovic et al. (2009)	Monocenter, prospective cohort	33.8 (0.0–451.9)	23.5 (0.0–77.1)		PPV = 5.9%, NPV = 97.6%, PLR = 1.6 (1.3–1.9%), NLR = 0.6 (0.4–0.8_	62.9% 60.4%	1st-2nd (6–15 GW)	1560	62	
			329.8 (22.3–2894.4)	213.9 (0.0–969.6)		PPV = 8%, NPV = 97.5%, PLR = 2.2 (1.7–2.7%), NLR = 0.6 (0.5–0.8)	51.6% 76.4%	2nd (20–25 GW)			
	Ghosh et al. (2013)	Monocenter, prospective cohort	<144 in 176/426 underweight women, 394/976 normal women, 68/192 overweight, 28/84 obese			PlGF <144 and EOPE: OR in obese/overweight women = 7.64 (5.34–10.12) OR in normal/underweight women = 2.95 (1.74–4.26)	n/a	2nd (20–22 GW)	1657		21
	Kumar et al. (2017)	Monocenter, observational, prospective cohort	37.31 ± 13.28, median = 34.2	30.42 ± 10.22, median = 34.2		OR = 3.57 (1.36–6.84)	n/a	1st (11–13 GW)	1206	208 (HDP)	74 (HDP)
	Kenny et al. (2014)	Multicenter, prospective, non-interventional cohort	977 (545–1649)	642 (347–1252)		n/a		2nd (14–16 GW)	5317	306	28
	Chaiyasit et al., 2022	Multicenter, prospective, non-interventional cohort	37.32 (27.54–50.06)	26.88 (18.89–237.61)		n/a		1st (11–13 GW)	7736	141	
ng/ml	Vieira et al. (2017)	Multicenter, prospective, cohort	1.01 (0.56–1.72) (normal BMI), 1.00 (0.55–1.70) (obese)	0.71 (0.41–1.68) (normal BMI), 0.66 (0.31–1.31) (obese)		OR = 1.25 (1.00–1.56) in normal BMI OR = 1.82 (1.37–2.42) in obese women	n/a	1st-2nd (10–16 GW)	3767	182	
MoM	Sonek et al. (2018)	Monocenter, prospective observational cohort	1.01 (0.81–1.27)	Late onset: 1.07 (0.84–1.28)	0.68 (0.38–1.17)	n/a		1st (11–13 GW)	1022	Late onset:46	13
	Hu et al. (2021)	Multicenter, prospective, population-based	0.99 (0.73–1.32)	Late onset: 0.83 (0.53–1.09) term PE	0.91 (0.63–1.18)	n/a		1st (11–13 GW)	8333	Late onset: 195	117
	Hanchard et al. (2020)	Monocenter, prospective cohort	1.09 (1.06–1.12)	0.95 (0.84–1.06)		OR = 0.258 (0.093–0.711)	n/a	1st-2nd (10–14 GW)	1086	55 (HDP)	
	Panaitescu et al. (2018)	Multicenter, prospective, observational	1.019 (0.559–1.832)	0.334 (0.198–0.556)		n/a		3rd (35–37 GW)	13078	272	
no value provided	O'Gorman et al. (2017)	Multicenter, prospective, non-interventional				n/a		1st (11–13 GW)	8297	239	76
	Boutin et al. (2018)	Monocenter, prospective cohort				n/a		1st (11–13 GW)	4420	232	30
	Chaemsaithong et al. (2019)	Monocenter, prospective, non-interventional cohort				n/a		1st (11–13 GW)	3982	41	

**Figure 1:**
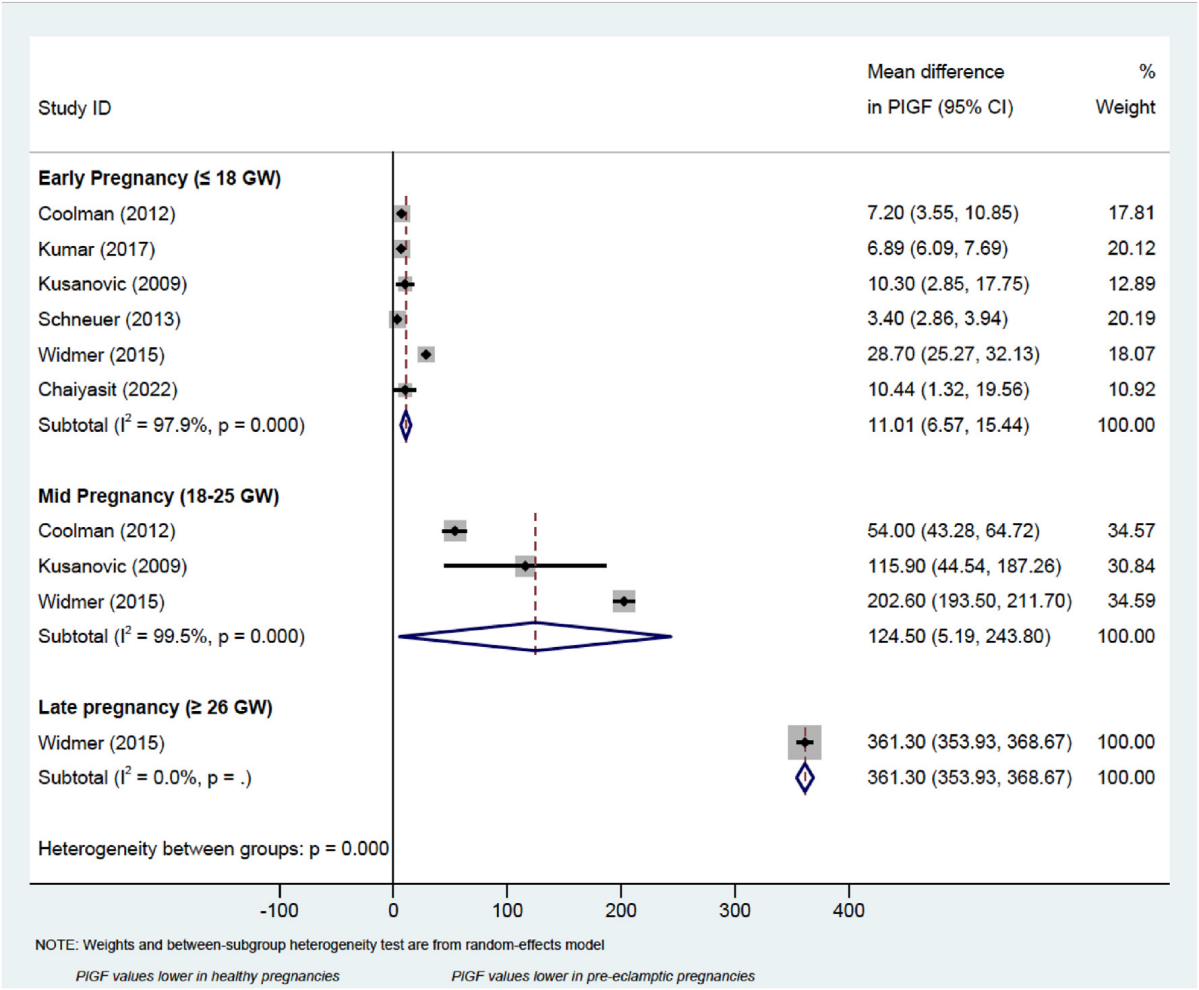
Forest plot of estimated mean difference in PlGF values between healthy pregnancies and those complicated by pre-eclampsia.

**Figure 2:**
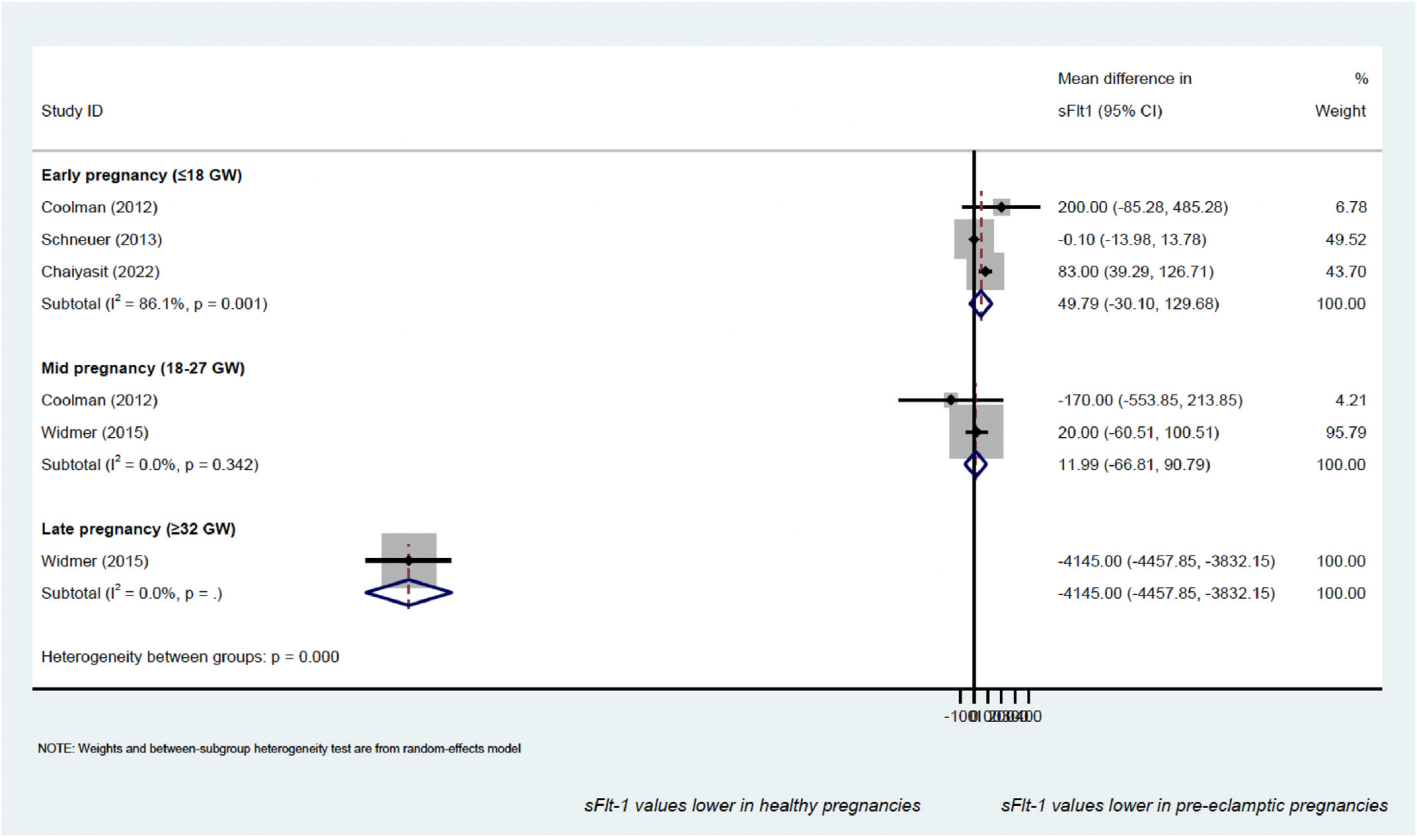
Forest plot of the mean difference in sFlt-1 between healthy pregnancies and those complicated by pre-eclampsia.

Meta-analysis was performed on studies reporting PlGF measurements throughout pregnancy, which were subdivided into early (before or at 18 gestational weeks), mid (between 18 and 26 gestational weeks), and late (at or after 26 gestational weeks), as shown in the forest plot (Figure 2). The mean difference in PlGF measurement (95% CI) between the healthy and pre-eclamptic groups increases as the pregnancy progresses, ranging from 11.01 (4.51, 17.91) in early pregnancy, 124.50 (5.19, 243.80) in mid pregnancy, up to 361.30 (353.93, 368.67) after 26 GW (Figure 1). Therefore, mean difference in PlGF levels between pre-eclampsia and healthy pregnancies, appear to increase as the gestational age progresses.

### 3.3 Pregnancy-associated plasma protein A (PAPP-A)

PAPP-A was measured in 12 [69,74,80,82,83,96,100,102,103,105,107,110] studies included in this systematic review, but only 10 studies provided numeric data of its serum concentration (Table S5). To study PAPP-A levels have high predictive value in early pregnancy, all the 10 studies focused on the first trimester. A total of 39,749 healthy pregnancies and 1,505 pre-eclamptic women had PAPP-A measured in their blood. In line with current evidence, PAPP-A measurements were lower in pre-eclamptic women in 8 studies.

### 3.4 Soluble Fms-Like Tyrosine Kinase-1 (sFlt-1)

As shown in Table S6, eight studies [68, 71, 72, 73, 78, 91, 105, 108] included in our systematic review provided values for this protein, but its measurements were not consistently associated with pre-eclampsia diagnosis or prediction, particularly in early pregnancy. sFlt-1 has been measured in 33,743 healthy and 1,219 pre-eclamptic women throughout pregnancy (from 10 up to 37 gestational weeks). Meta-analysis has been performed with the aim of comparing sFlt-1 levels measured in pregnant women in early (before 18 GW), mid (between 18 and 27 GW) and late (later than 32 GW) pregnancy, as shown in Figure 2.

### 3.5 Beta human chorionic gonadotropin (beta-hCG)

A total of 9 studies [46, 74, 79, 96, 97, 102, 105, 109, 110] focused on beta-hCG blood measurements during the first and early second (no later than 20 gestational weeks) trimesters, and samples from 31,627 healthy and 2,133 hypertensive pregnant women have been analyzed (Table S7). When reported, serum levels were very similar between the control and the disease groups. No clear association between beta-hCG concentrations and the development of pregnancy hypertension can be drawn from the studies included in this systematic review.

### 3.6 sFlt-1/PlGF

In our systematic review the ratio between sFlt-1 and PlGF was increased in the 766 pre-eclamptic women as compared to the control group (24,924 healthy pregnancies) in a total of four studies [46, 68, 72, 78]. Three studies published data regarding both early and late onset pre-eclampsia (Table S8).

### 3.7 Leptin

In this systematic review, leptin levels were measured in three studies [70, 85, 99], for a total of 8,523 healthy and 511 pre-eclamptic women (Table S9). Mean serum levels were comparable among groups, proving to be in two cases slightly lower (19 ng/mL vs 25 ng/mL, and 63.38 ng/mL vs 63.64 ng/mL), and in one case mildly higher (11.3 ng/mL vs 9.3 ng/mL) in pre-eclampsia as compared to normal pregnancies. All the studies included in this systematic review focused on second trimester measurements, when leptin levels appear to be increased in pre-eclamptic patients [111].

### 3.8 Soluble endoglin (sEng)

Three studies [68, 77, 87] included in this systematic review measured leptin levels in 10256 healthy and 442 pre-eclamptic pregnancies from the first up to the third trimester of gestation (Table S10). Serum levels of Endoglin have been reported to be consistently higher in women with a diagnosis of pre-eclampsia.

### 3.9 Alpha fetoprotein (AFP)

In three of the included studies [82, 109, 110] AFP maternal levels were higher in the blood of healthy women as compared to the ones developing pre-eclampsia. However, according to Farzanehet et al. [97] and Boutin et al. [105], the levels of the same proteins are increased in pre-eclampsia when measured in the early second trimester (15–18 gestational weeks) and between the first and the second (11–13 GW) respectively. Details of AFP measurements can be found in Supplementary Table S11.

### 3.10 Uric Acid (UA)

As shown in Supplementary Table S12, UA levels were reported to be consistently higher in women with pre-eclampsia as compared to healthy pregnancies in all the included studies [88, 101, 106]. Measurements were performed from the first trimester (10 GW) up to the late second trimester (24 GW).

### 3.11 Unconjugated estriol (UE3)

Three studies [88, 97, 109] only considered unconjugated estriol (UE3) measurement and their potential correlation with pre-eclampsia development in the second trimester (Supplementary Table S13). Only two of them reported numerical values, and in both cases pre-eclamptic women had slightly lower serum levels.

### 3.12 Other biomarkers

Sixty-two additional biomarkers have been assessed among twenty-one studies (Table S14).

Plasminogen activator inhibitor-2 (PAI-2) is a protein not normally detected in human blood under physiological conditions [112]. Its levels increase during pregnancy when it becomes measurable [113]. PAI-2 concentration was found to be lower in pre-eclampsia as compared to normal pregnancy in one study only, where samples were taken during the second trimester [70]. On the other hand, no link between PAI-2 levels and pre-eclampsia development were established in the first trimester [72]. Hemoglobin A1C (HbA1c) is routinely measured at the booking appointment in the blood of pregnant women at high risk of developing gestational diabetes [114]. It was measured in two studies, which showed a higher incidence of pre-eclampsia in women with raised values [75, 90]. C-reactive Protein (CRP) is a routinely measured blood biomarker of inflammation and infection [115]. Its levels appeared to be raised in future pre-eclamptic patients when measured in early pregnancy in both studies part of this systematic review [76, 99]. B-type natriuretic peptide (BNP) is the most widely used indicator of heart failure worldwide [116]. When measured in early pregnancy, its blood concentration was higher in pre-eclamptic women and lower in pregnancies not complicated by hypertensive disorders [99]. However, the same correlation was not found in a study by Vieira et al. [77]. The same two aforementioned studies measured cystatin C, a promising biomarker for glomerular filtration rate [117]: its levels were higher in obese women prone to develop pre-eclampsia [77] and slightly higher in pre-eclamptic nulliparous women (without considering their BMI) [99].

As shown in Supplementary Table S14, further biomarkers taken into consideration were routine blood parameters like platelets, 25-hydroxyvitamin, HDL cholesterol, hematocrit, mean corpuscular volume, red blood cell distribution width, mean platelet volume, antithrombin III, haptoglobin, iron, transferrin, ferritin, total proteins, albumin, calcium, magnesium, sodium, potassium, urea nitrogen, creatinine, lactate dehydrogenase (LDH), aspartate transaminase and alanine transaminase (AST/ALT) ratio, gamma-glutamyl transpeptidase (GGT), alkaline phosphatase (ALP), estimated glomerular filtration rate (eGFR). Hormones like progesterone, free estriol, and estriol/progesterone ratio were also assessed. Other molecules measured in the blood of pregnant women include tumor necrosis factor alpha (TNF alpha), leptin receptor, transforming growth factor beta (TGF-beta), angioprotein 1 (Ang-1) and angioprotein 2 (Ang-2), adiponectin, atrial natriuretic peptide (ANP), angiogenin, elafin, intracellular adhesion molecule-1 (ICAM-1), interleukin-1 receptor antagonist 1 (IL-1Ra), tissue inhibitor of metalloproteinase-1 (TIMP-1), interferon gamma (INF-gamma), selenoprotein P, plasma 1 (SEPP-1), placental Protein 13 (PP13), soluble vascular endothelial growth factor receptor-1 (sVEGFR-1), sLIGHT, Inhibin A, and death associated protein kinase 1 (DAPK-1).

### 3.13 Assessment of publication bias

Publication bias was assessed using Begg’s test (there were too few numbers of studies to meaningfully use funnel plots). When there were enough studies (>2), publication bias was assessed using Begg’s test (not enough studies were identified for a meaningful use of funnel plots). For PlGF, the six studies carried out in semester one, and four studies in semester two, no significant publication bias was identified (p = 0.624 and p = 0.602 respectively). For the three studies contributing to the meta-analysis of sFlt-1 (measurements taken in the first trimester), again no evidence of publication bias was found (p = 0.602).

Study quality assessment was performed using the Newcastle-Ottawa Scale (NOS) for non-randomized studies in meta-analysis [18], as shown in Supplementary Material (Table S2). Overall, high quality studies were included in this systematic review with meta-analysis. Comparability was assessed awarding one star if the study adjusted for maternal age, gestational age, race, or BMI. Unadjusted analyses were performed in some of the three studies, and this accounted for a drop in marks. Overall, all the included studies scored between 6 and 7 stars.

## 4 Discussion

### 4.1 Main findings

The outcomes of this systematic review with meta-analysis suggests that PlGF may be a useful diagnostic tool for pre-eclampsia diagnosis in the third trimester. However, its predictive value in early pregnancy is not high enough for an early diagnosis, and it generally becomes more reliable as the pregnancy progresses. Results from sFlt-1 meta-analysis were inconsistent. Other biomarkers have been included in this systematic review, but further meta-analyses have not been performed due to lack of sufficiently homogenous data. Consistent results among studies have been highlighted for PAPP-A, leptin, sEng, sFlt-1/PlGF, hemoglobin, CRP, and cystatin.

### 4.2 Strengths and limitations

A search of multiple databases together with a comprehensive search strategy are among the main strengths of this systematic review. Furthermore, no language, date, or gestational week restrictions have been applied. Publication bias and study quality were both assessed, and a meta-analysis was performed for one biomarker. High quality and prospective studies only have been included in this systematic review.

However, meta-analysis was not performed for the remaining biomarkers due to lack of homogenous data. Because of the high volume of research, we decided to analyze blood biomarkers only, thus not considering ultrasound, urinary or salivary molecules. Also, another important limitation of this systematic review was the lack of homogenous data due to the use of different laboratory methodologies and different kits throughout the included studies. Furthermore, the effect of different confounding factors among the study populations must be considered when interpreting the results. Due to the great variety of study designs and methods used, confounding factors could not be assessed when analyzing the results. Their effect should be statistically investigated in a larger single study using single patient data. Finally, no individual patient data was extracted and consequently no clinical difference between single patients can be appreciated.

### 4.3 Interpretations

To the best of our knowledge, this is the first systematic review with meta-analysis that has no limit in terms of language or year of publication and that has investigated all blood biomarkers for the prediction or diagnosis of pre-eclampsia. The systematic review findings indicate that we currently lack an ideal biomarker for the early detection of pre-eclampsia. The use of PlGF to predict pre-eclampsia in the third trimester has been confirmed by this meta-analysis, but further studies are needed to investigate its role as a predictor of pre-eclampsia in early pregnancy. Biologically, PlGF is a proangiogenic molecule with a predominant role between the second and third trimester of pregnancy (26–30 weeks of gestation) [23]. At this time, PlGF is responsible for inducing systemic vasodilation in the placenta bed by stimulating cells to produce prostacyclin and nitric oxide, hence transforming placental vessels from high-resistance to low-resistance vessels [118]. Consequently, low levels of PlGF in late pregnancy would result in placental hypoperfusion predisposing to pregnancy complications, in particular, pre-eclampsia [23].

## 5 Conclusions

Despite a better understanding of its pathophysiology, pre-eclampsia remains one of the most severe pregnancy complications. Several biomarkers have been evaluated throughout the years with the aim of a more accurate and earlier prediction of the disease. So far, PlGF and sFlt-1 are the most used in clinical practice, even if their specificity and sensitivity do not justify a default implementation into guidelines to screen all pregnant women. Their role is limited to rule-out a diagnosis of pre-eclampsia in women presenting with symptoms highly suggestive of the disease. Therefore, their current application is not useful for an early prediction of pre-eclampsia. Novel lines of research are needed to identify molecules able to detect pre-eclampsia in a timely manner and which have a predictive value high enough to justify the costs associated with routine measurements in the general pregnant population.

## Declarations

### Author contribution statement

Bee Kang Tan: Conceived and designed the experiments; Analyzed and interpreted the data.

Marianna Danielli: Performed the experiments; Analyzed and interpreted the data; Wrote the paper.

Roisin C. Thomas and Jiamiao Hu: Performed the experiments.

Clare L. Gillies and Kamlesh Khunti: Analyzed and interpreted the data.

### Funding statement

Bee Kang Tan was supported by the John and Lucille Van Geest Foundation and the Medical Research Council (MR/R020981/2).

Kamlesh Khunti was supported by the National Institute for Health Research.

### Data availability statement

Data included in article/supp. material/referenced in article.

### Declaration of interest’s statement

The authors declare no conflict of interest.

### Additional information

No additional information is available for this paper.

## Appendix A Supplementary data

HELIYON-D-22-11307 Supplementary HELIYON-D-22-11307 Supplementary Material Revise 29092022 HELIYON-D-22-11307 Supplementary HELIYON-D-22-11307 Supplementary Material Revise 29092022
