# Supplementary material for: Blood biomarkers to predict the onset of pre-eclampsia: A systematic review and meta-analysis
Source: Heliyon. 2022 Nov 4;8(11):e11226. doi: 10.1016/j.heliyon.2022.e11226 (PMC9649987; doi:10.1016/j.heliyon.2022.e11226)
Supplement: HELIYON-D-22-11307 Supplementary HELIYON-D-22-11307 Supplementary Material Revise 29092022 [file mmc1.docx]

**Supplementary Figure S1. Medline Search Strategy.**

exp Hypertension, Pregnancy-Induced/ OR (pregnan* adj3 hypertens*).tw,kw. OR maternal hypertension.tw,kw. OR gestational hypertension.tw,kw. OR eclamp*.tw,kw. OR HELLP.tw,kw. OR "h?emolysis elevated liver enzymes low platelet count*".tw,kw. OR preeclamp*.tw,kw. OR pre-eclamp*.tw,kw. OR (pregnan* adj3 tox?emi*).tw,kw. OR (gestation* adj3 tox?emi*).tw,kw. OR "EPH complex*".tw,kw. OR Gestosis.tw,kw. OR "EPH tox?emia*".tw,kw. OR (Proteinuria adj3 Hypertension).tw,kw.

AND

Biomarkers/an, bl [Analysis, Blood] OR biomarker*.tw,kw. OR bio-marker*.tw,kw. OR marker*.tw,kw. OR Biological Factors/ OR biological factor*.tw,kw.

**Supplementary Table S1. PRISMA 2009 Checklist.**

| **Section/topic** | **#** | **Checklist item** | **Reported on page #** |
| --- | --- | --- | --- |
| **TITLE** | | |  |
| Title | 1 | Identify the report as a systematic review, meta-analysis, or both. | 1 |
| **ABSTRACT** | | |  |
| Structured summary | 2 | Provide a structured summary including, as applicable: background; objectives; data sources; study eligibility criteria, participants, and interventions; study appraisal and synthesis methods; results; limitations; conclusions and implications of key findings; systematic review registration number. | 2 |
| **INTRODUCTION** | | |  |
| Rationale | 3 | Describe the rationale for the review in the context of what is already known. | 3, 4 |
| Objectives | 4 | Provide an explicit statement of questions being addressed with reference to participants, interventions, comparisons, outcomes, and study design (PICOS). | 4 |
| **METHODS** | | |  |
| Protocol and registration | 5 | Indicate if a review protocol exists, if and where it can be accessed (e.g., Web address), and, if available, provide registration information including registration number. | 4 |
| Eligibility criteria | 6 | Specify study characteristics (e.g., PICOS, length of follow-up) and report characteristics (e.g., years considered, language, publication status) used as criteria for eligibility, giving rationale. | 5 |
| Information sources | 7 | Describe all information sources (e.g., databases with dates of coverage, contact with study authors to identify additional studies) in the search and date last searched. | 4, 5 |
| Search | 8 | Present full electronic search strategy for at least one database, including any limits used, such that it could be repeated. | Supplementary material (Figure S1) |
| Study selection | 9 | State the process for selecting studies (i.e., screening, eligibility, included in systematic review, and, if applicable, included in the meta-analysis). | 4-6, Table 2 |
| Data collection process | 10 | Describe method of data extraction from reports (e.g., piloted forms, independently, in duplicate) and any processes for obtaining and confirming data from investigators. | 4, 5 |
| Data items | 11 | List and define all variables for which data were sought (e.g., PICOS, funding sources) and any assumptions and simplifications made. | 5 |
| Risk of bias in individual studies | 12 | Describe methods used for assessing risk of bias of individual studies (including specification of whether this was done at the study or outcome level), and how this information is to be used in any data synthesis. | 5, 6 Supplementary material (Table S2) |
| Summary measures | 13 | State the principal summary measures (e.g., risk ratio, difference in means). | 6 |
| Synthesis of results | 14 | Describe the methods of handling data and combining results of studies, if done, including measures of consistency (e.g., I^2^) for each meta-analysis. | 6 |

Page 1 of 2

| **Section/topic** | **#** | **Checklist item** | **Reported on page #** |
| --- | --- | --- | --- |
| Risk of bias across studies | 15 | Specify any assessment of risk of bias that may affect the cumulative evidence (e.g., publication bias, selective reporting within studies). | 5-6, Supplementary material (Figure S2) |
| Additional analyses | 16 | Describe methods of additional analyses (e.g., sensitivity or subgroup analyses, meta-regression), if done, indicating which were pre-specified. | 6 |
| **RESULTS** | | |  |
| Study selection | 17 | Give numbers of studies screened, assessed for eligibility, and included in the review, with reasons for exclusions at each stage, ideally with a flow diagram. | 10-11, Table 2 |
| Study characteristics | 18 | For each study, present characteristics for which data were extracted (e.g., study size, PICOS, follow-up period) and provide the citations. | 12, Supplementary material (Table S3) |
| Risk of bias within studies | 19 | Present data on risk of bias of each study and, if available, any outcome level assessment (see item 12). | 21-22, Supplementary material (Table S2) |
| Results of individual studies | 20 | For all outcomes considered (benefits or harms), present, for each study: (a) simple summary data for each intervention group (b) effect estimates and confidence intervals, ideally with a forest plot. | 13-21  Table 3, Supplementary material (Tables S5-S13) |
| Synthesis of results | 21 | Present results of each meta-analysis done, including confidence intervals and measures of consistency. | 12, 18, Figures 1-2 |
| Risk of bias across studies | 22 | Present results of any assessment of risk of bias across studies (see Item 15). | 21-22, Table S2 |
| Additional analysis | 23 | Give results of additional analyses, if done (e.g., sensitivity or subgroup analyses, meta-regression [see Item 16]). | n/a |
| **DISCUSSION** | | |  |
| Summary of evidence | 24 | Summarize the main findings including the strength of evidence for each main outcome; consider their relevance to key groups (e.g., healthcare providers, users, and policy makers). | 22 |
| Limitations | 25 | Discuss limitations at study and outcome level (e.g., risk of bias), and at review-level (e.g., incomplete retrieval of identified research, reporting bias). | 22-23 |
| Conclusions | 26 | Provide a general interpretation of the results in the context of other evidence, and implications for future research. | 23-24 |
| **FUNDING** | | |  |
| Funding | 27 | Describe sources of funding for the systematic review and other support (e.g., supply of data); role of funders for the systematic review. | 24-25 |

**Supplementary Table S2. Newcastle-Ottawa Scale (NOS) for nonrandomized studies**

| **Study** | **Selection** | | | | **Comparability** | **Outcome** | | | **Total stars** |
| --- | --- | --- | --- | --- | --- | --- | --- | --- | --- |
|  | Representativeness of exposed cohort | Selection of non-exposed cohort | Ascertainment of exposure | Outcome of interest not present at start of study | Comparability of cohorts on the basis of the design or analysis | Assessment of outcome | Follow-up long enough for outcomes to occur | Adequacy of follow-up of cohorts |  |
| Widmer et al., 2015 | ★ | ★ | ★ | ★ | ★ | ★ | ★ |  | **7** |
| O'Gorman et al., 2017 | ★ | ★ | ★ | ★ | ★ | ★ | ★ |  | 7 |
| Clausen et al., 2002 | ★ | ★ | ★ | ★ |  | ★ | ★ |  | 6 |
| Honigberg et at., 2016 | ★ | ★ | ★ | ★ | ★ | ★ | ★ |  | 7 |
| Coolman et al., 2012 | ★ | ★ | ★ | ★ | ★ | ★ | ★ |  | 7 |
| Schneuer et al., 2014 | ★ | ★ | ★ | ★ | ★ | ★ | ★ |  | 7 |
| Honarjoo et al., 2019 | ★ | ★ | ★ | ★ | ★ | ★ | ★ |  | 7 |
| Mañé et al., 2019 | ★ | ★ | ★ | ★ | ★ | ★ | ★ |  | 7 |
| De Jonge et al., 2011 | ★ | ★ | ★ | ★ | ★ | ★ | ★ |  | 7 |
| Vieira et al., 2017 | ★ | ★ | ★ | ★ | ★ | ★ | ★ |  | 7 |
| Herraiz et al., 2018 | ★ | ★ | ★ | ★ |  | ★ | ★ |  | 6 |
| Asvold et al., 2014 | ★ | ★ | ★ | ★ | ★ | ★ | ★ |  | 7 |
| Yung et al., 2019 | ★ | ★ | ★ | ★ | ★ | ★ | ★ |  | 7 |
| Boutin et al., 2017 | ★ | ★ | ★ | ★ |  | ★ | ★ |  | 6 |
| Kenny et al., 2014 | ★ | ★ | ★ | ★ | ★ | ★ | ★ |  | 7 |
| Schneuer et al., 2013 | ★ | ★ | ★ | ★ | ★ | ★ | ★ |  | 7 |
| Rezk et al., 2018 | ★ | ★ | ★ | ★ | ★ | ★ | ★ |  | 7 |
| Sharma et al., 2018 | ★ | ★ | ★ | ★ |  | ★ | ★ |  | 6 |
| Boutin et al., 2018 | ★ | ★ | ★ | ★ |  | ★ | ★ |  | 6 |
| Chaemsaithong et al., 2019 | ★ | ★ | ★ | ★ | ★ | ★ | ★ |  | 7 |
| Boutin et al., 2020 | ★ | ★ | ★ | ★ |  | ★ | ★ |  | 6 |
| Sonek et al., 2018 | ★ | ★ | ★ | ★ | ★ | ★ | ★ |  | 7 |
| Chen et al., 2021 | ★ | ★ | ★ | ★ | ★ | ★ | ★ |  | 7 |
| Barjaktarovic et al., 2019 | ★ | ★ | ★ | ★ | ★ | ★ | ★ |  | 7 |
| Stratieva et al., 2010 | ★ | ★ | ★ | ★ | ★ | ★ | ★ |  | 7 |
| Wu et al., 2017 | ★ | ★ | ★ | ★ | ★ | ★ | ★ |  | 7 |
| Tasleem et al., 2016 | ★ | ★ | ★ | ★ |  | ★ | ★ |  | 6 |
| Gonen et al., 2008 | ★ | ★ | ★ | ★ |  | ★ | ★ |  | 6 |
| Kusanovic et al., 2009 | ★ | ★ | ★ | ★ | ★ | ★ | ★ |  | 7 |
| Hu et al., 2021 | ★ | ★ | ★ | ★ | ★ | ★ | ★ |  | 7 |
| Chaiyasit et al., 2022 | ★ | ★ | ★ | ★ | ★ | ★ | ★ |  | 7 |
| Massé et al., 1993 | ★ | ★ | ★ | ★ |  | ★ | ★ |  | 6 |
| Boyle et al., 2016 | ★ | ★ | ★ | ★ | ★ | ★ | ★ |  | 7 |
| Mañé et al., 2017 | ★ | ★ | ★ | ★ | ★ | ★ | ★ |  | 7 |
| Panaitescu et al., 2018 | ★ | ★ | ★ | ★ | ★ | ★ | ★ |  | 7 |
| Yue et al., 2020 | ★ | ★ | ★ | ★ | ★ | ★ | ★ |  | 7 |
| Ghosh et al., 2013 | ★ | ★ | ★ | ★ | ★ | ★ | ★ |  | 7 |
| Hirashima et al., 2018 | ★ | ★ | ★ | ★ |  | ★ | ★ |  | 6 |
| Gybel-Brask et al., 2014 | ★ | ★ | ★ | ★ | ★ | ★ | ★ |  | 7 |
| Stonek et al., 2007 | ★ | ★ | ★ | ★ |  | ★ | ★ |  | 6 |
| Hanchard et al., 2020 | ★ | ★ | ★ | ★ | ★ | ★ | ★ |  | 7 |
| Kumar et al., 2017 | ★ | ★ | ★ | ★ |  | ★ | ★ |  | 6 |
| Farzaneh et al., 2019 | ★ | ★ | ★ | ★ | ★ | ★ | ★ |  | 7 |
| Kiely et al., 2016 | ★ | ★ | ★ | ★ | ★ | ★ | ★ |  | 7 |

**Supplementary Table S3. Table of Study Characteristics.**

| **Study** | **Country** | **Study design** | **Number of participants (total)** | **Number of participants (PE)** | **Mean age (years)** | **Mean BMI (kg/m2)** | **Ethnicity** | **Serum/Plasma** | **analysis** | **Exclusion criteria** | **Inclusion criteria** | **Gestational Week (GW)** |
| --- | --- | --- | --- | --- | --- | --- | --- | --- | --- | --- | --- | --- |
| Widmer et al., 2015 | international | prospective, multicenter | 5121 | 198 | 23.1 | 21.7 | mixed | serum | ELISA | renal disease, proteinuria | women at risk of PE | <20, 23-27, 32-35 |
| O'Gorman et al., 2017 | international | Multicenter, prospective, non-interventional | 8536 | 239 | 31.5 | 24.5 | mixed | serum | DELFIA® Xpress kits | multiple pregnancy, aneuploidy, foetal abnormalities, TOP, miscarriages | maternal age ≥ 18 years, no serious mental illness or learning difficulty, delivery ≥ 24 GW | 11-13 |
| Clausen et al., 2002 | Norway | Monocenter, prospective | 2190 | 71 | 29 | 23.4 | Caucasian | serum | ELISA, competitive radioimmunoassay | chronic hypertension, renal disease, diabetes, GDM, multiple pregnancy | previously healthy women | 17-19 |
| Honigberg et at., 2016 | USA | Multicenter, prospective, | 2355 | 202 | 31.2 | 25.8 | mixed | serum | ARCHITECT immunoassays | multiple pregnancy (triplets or more) | maternal age > 18 years | 10, 18, 26, 35 |
| Coolman et al., 2012 | The Netherlands | Monocenter, population-based prospective cohort | 7519 | 167 | 29.5 | 23.8 | mixed | plasma | ELISA, immunoelectrochemoluminescence assay | stillbirth | delivery > 22 GW | <18, 18-25 |
| Schneuer et al., 2014 | Australia | monocenter, population-based prospective cohort | 4621 | 163 | 32.9 | n/a | n/a | serum | ELISA | multiple pregnancy, abortion, fetus with major congenital defect | singleton live births | 10-13 |
| Honarjoo et al., 2019 | Iran | Monocenter, prospective observational cohort | 4605 | 333 | 28.5 | 24.5 | n/a | serum | n/a | multiple pregnancy, structural defects, visible anomaly, Down syndrome, home delivery, drugs, narcotics, cigarettes or alcohol use, pregnancy disease | combined fetal anomaly screening tests performed | 10-13 |
| Mañé et al., 2019 | Spain | Monocenter, prospective | 1882 | 68 | 33.4 | 24.8 | mixed | plasma | high-performance liquid chromatography | multiple pregnancy, pre-existing diabetes (type 1 or 2), miscarriage, TOP, lost at follow-up | maternal age >18 years | 10-13 |
| De Jonge et al., 2011 | The Netherlands | Multicenter, prospective, cohort | 5816 | 92 | 30.3 | 24.4 | mixed | plasma | immunoturbidimetric assay | multiple pregnancy, high CRP, pre-existing hypertension, pre-existing diabetes, high cholesterol, SLE, any chronic heart condition | singleton live births | <18 |
| Vieira et al., 2017 | international | Multicenter, prospective, cohort | 3949 | 182 | 28.7 | 25.5 | mixed | plasma | immunoassays | high risk pregnancies, underlying medical conditions, three previous miscarriages or TOP, major anomaly, abnormal karyotype | healthy, nulliparous, singleton pregnancy | 14-16 |
| Herraiz et al., 2018 | Spain | Monocenter, prospective, cohort | 5601 | 236 | 31.2 | 24.3 | mixed | serum | automated assay system | multiple pregnancy, chromosomal anomalies, major malformations or congenital infections, unknown pregnancy outcome and lack of informed consent | viable singleton pregnancy | 24-28 |
| Asvold et al., 2014 | Norway | Monocenter, prospective | 2405 | 105 | 33.2 | 22.7 | n/a | serum | n/a | multiple pregnancy | IVF pregnancy | 1+5 |
| Yung et al., 2019 | Australia | Monocenter, prospective | 2048 | 113 | 32.6 | 24.8 | mixed | serum | ELISA | abnormal mid-trimester foetal morphology, non-speaking English | singleton pregnancy, age >18 | 36 |
| Boutin et al., 2017 | Canada | Monocenter, prospective, cohort | 4739 | 232 | 28.9 | 25 | mixed | serum | automated immunofluorescent assay | Multiple pregnancy, miscarriages, TOP | nulliparous women | 13 |
| Kenny et al., 2014 | international | Multicenter, prospective | 5623 | 306 | 28.5 | n/a | mixed | plasma | immunoassays | miscarriage, ineligible, lack of consent, closure of recruitment | nulliparous women | 14-16 |
| Schneuer et al., 2013 | Australia | Monocenter, prospective | 2749 | 68 | 32.7 | n/a | mixed | serum | ELISA, automated immunoassay | multiple pregnancy, abortion, past infant with a major congenital anomaly | women attending 1st trimester USS | 10-14 |
| Rezk et al., 2018 | Egypt | Monocenter, prospective, longitudinal | 9522 | 286 | 31.1 | 26 | mixed | serum | calorimetric technique | smoking, alcohol consumption, high risk of PE, pre-existing medical disorders | low or moderate risk of PE | 10-12, 18-20 |
| Sharma et al., 2018 | India | Monocenter, prospective | 2000 | 45 | 24.5 | 20.8 | n/a | serum | ELISA, chemiluminescence based auto analyzer | multiple pregnancy, lost to follow-up, TOP<28 GW, medical conditions | healthy, singleton pregnancy | 11-13 |
| Boutin et al., 2018 | Canada | Monocenter, prospective cohort | 4652 | 232 | 28.9 | 25 | mixed | serum | immunofluorescence assays | multiple pregnancy, TOP, aspirin use | nulliparous, singleton live pregnancies | 11-13 |
| Chaemsaithong et al., 2019 | international | Monocenter, prospective, non-interventional cohort | 4023 | 41 | 32.6 | 21.5 | mixed | serum | automated analyzers | unconsciousness, severe illness, learning difficulties, serious mental illness, major fetal aneuploidy, or abnormality | maternal age ≥ 18 years, singleton pregnancy and live fetus | 11-13 |
| Boutin et al., 2020 | Canada | Monocenter, prospective cohort | 4659 | 225 | 28.7 | 23.8 | mixed | serum | immunofluorescence assays | multiple pregnancy, foetal death, TOP, foetal anomaly, aspirin use | nulliparous pregnant women, live fetus | 11-13 |
| Sonek et al., 2018 | USA | Prospective observational cohort | 1068 | 46 | 28.9 | 27.4 | mixed | serum | fluorometry sandwich immunoassays | multiple pregnancy, fetal congenital anomalies, delivery < 20 GW | singleton live births | 11-13 |
| Chen et al., 2021 | China | Monocenter, prospective, cohort | 1041 | 31 | 29.5 | 20.5 | Asian | venous blood | automated analyzers | IVF, multiple pregnancy, miscarriage, hepatitis, chronic nephritis, rheumatoid arthritis, SLE | singleton live births | <18 |
| Barjaktarovic et al., 2019 | The Netherlands | Monocenter, prospective, cohort | 7754 | 165 | 29.6 | 24.7 | mixed | serum | chemiluminescence immunometric assay | multiple pregnancy, no hCG measurement, IVF, pre-existing hypertension, non-availability of data | singleton live births | 14.4 |
| Stratieva et al., 2010 | UK | Monocenter, prospective | 8366 | 165 | n/a | n/a | n/a | serum | DELFIA® Xpress kits | missing outcome data, major fetal defect, aneuploidy, fetal death, miscarriage, TOP | singleton live births | 11-13 |
| Wu et al., 2017 | China | Multicenter, prospective | 2434 | 1034 | 30.7 | n/a | Asian | venous blood | real-time PCR | multiple pregnancy, pre-existing chronic hypertension or other disease | singleton live birth | >28 |
| Tasleem et al., 2016 | Pakistan | Monocenter, prospective | 1221 | 134 | 27.5 | 25.8 | n/a | serum | ELISA | multiple pregnancy, previous PE, any pre-existent disease | singleton live births | 16 |
| Gonen et al., 2008 | Israel | Multicenter, prospective, longitudinal, observational | 1366 | 20 | 30 | 22.6 | mixed | serum | ELISA | noncompliance with protocol, miscarriage | singleton live births | 6-10, 16-20, 24-28 |
| Kusanovic et al., 2009 | Chile | Monocenter, prospective cohort | 1622 | 62 | 26 | 24.4 | n/a | plasma | ELISA | multiple pregnancy, major fetal anomaly | singleton live births | 6-15, 20-25 |
| Hu et al., 2021 | China | Multicenter, prospective, population-based | 10899 | 312 | 29.5 | 22 | Asian | serum | immunoassays | inability to give consent, TOP, major foetal abnormalities, miscarriage, foetal death | singleton pregnancy, age >18, maternal Chinese ethnicity | 11-13 |
| Chaiyasit et al., 2022 | international | Multicenter, prospective cohort | 7877 | 141 | 32.6 | 21.6 | Asian | serum | immunoassays | n/a | singleton live births | 11-13 |
| Massé et al., 1993 | Canada | Monocentre, prospective, cohort | 1366 | 109 | 26.1 | 21.9 | n/a | plasma/serum | nephelometry, refractometry, radioimmunoassay | maternal disease, first attendance > 20 GW, transient hypertension (delivery / early postpartum), abortion | nulliparous pregnant women | 15-24 |
| Boyle et al., 2016 | international | Multicentre, prospective | 1710 | 73 | 30.3 | 24.8 | mixed | serum | ELISA | n/a | n/a | 15 |
| Mañé et al., 2017 | Spain | Monocentre, prospective | 1228 | 47 | 32.6 | 25.4 | mixed | n/a | high-performance liquid chromatography | multiple pregnancy, pre-existing DM, TOP, miscarriage, unavailable data | maternal age > 18 years | ≤12 |
| Panaitescu et al., 2018 | UK | Multicenter, prospective, observational | 14450 | 272 | 32.2 | n/a | mixed | serum | automated biochemical analyzer | multiple pregnancy | singleton live births | 35-37 |
| Yue et al., 2020 | China | Monocentre, prospective | 12124 | 560 | 29.1 | 20.9 | Asian | serum | chemiluminescence immunometric assay | incomplete maternal/infant records, diabetes, hyperlipidemia, hypertension, cardiovascular disease, metabolic syndrome, history of severe systemic disease, untreated endocrine disease | singleton live births | 14-20 |
| Ghosh et al., 2013 | India | Monocentric, prospective cohort | 1677 | 21 | n/a | n/a | Asian | serum | ELISA | multiple pregnancy, fetal abnormalities | singleton live births | 20-22 |
| Hirashima et al., 2018 | Japan | Monocentre, prospective, cohort | 1724 | n/a | n/a | n/a | n/a | serum | ELISA | multiple pregnancy | singleton live births | 27-31 |
| Gybel-Brask et al., 2014 | Denmark | Monocentre, prospective, cohort | 1179 | 12 | 31 | 23.6 | n/a | serum | ELISA | multiple pregnancy | maternal age > 18 years | 12, 20, 25, 32 |
| Stonek et al., 2007 | Austria | Monocenter, prospective, controlled, open, | 1652 | 268 | 23.9 | 23.8 | Caucasian | venous blood | PCR | multiple pregnancy, failure of DNA extraction and/or failure of PCR, abortion, TOP | singleton live births | 12 |
| Hanchard et al., 2020 | Australia | Monocenter, prospective cohort | 1141 | 55 (HDP) | 31 | n/a | mixed | serum | DELFIA® Xpress kits | miscarriage, foetal death, TOP, essential hypertension, foetal anomalies, current treatment with aspirin, clexane or antihypertensive medications | consent for a transvaginal scan, singleton live pregnancy, embryo's crown–rump length (CRL) ≤ 45 mm, availability of pregnancy outcome data | 10-14 |
| Kumar et al., 2017 | India | Monocentric, prospective, observational cohort | 1725 | 208 (HDP) | 24.4 | 20.8 | n/a | serum | ELISA | multiple pregnancy, thrombophilia, aspirin use | singleton live births | 11-13 |
| Farzaneh et al., 2019 | Iran | Monocentric, prospective, cohort | 1713 | 151 | 32 | n/a | n/a | serum | electrochemiluminescence | multiple pregnancy, fetal anomaly, pre-existing maternal disease | singleton live births | 15-18 |
| Kiely et al., 2016 | Ireland | Monocenter, prospective | 1768 | 68 | 30.5 | 24.9 | mixed | serum | liquid chromatography–tandem mass spectrometry | high risk of PE, SGA baby, spontaneous preterm birth because of underlying medical conditions, previous cervical knife cone biopsy, ≥ TOP / miscarriages, PROM, major fetal anomaly / abnormal karyotype, aspirin use, cervical cerclage | low-risk pregnancy, nulliparity, no previous pregnancy >20 GW | 15 |

**Supplementary Table S4. Biomarkers analyzed in each study.**

| **Study** | **biomarker (unit of measurement)** | **biomarker value in healthy pregnancies** | **biomarker value in women with PE** | |
| --- | --- | --- | --- | --- |
|  |  |  | **any GW** | **⩽34 weeks (early onset PE)** |
| Widmer et al., 2015 | sEng(ng/ml) | 5.0 (3.9–6.4) | 5.5 (4.3–7.6) | 5.8 (4.3–8.4) |
|  | sFlt-1(pg/ml) | 2230 (1490–3340) | 1890 (1210–2840) | 2030 (1300–2930) |
|  | PlGF(pg/ml) | 84.6 (35.4–167.8) | 55.9 (23.5–117.4) | 59.9 (24.8–122.2) |
|  | sFlt-1/PlGF | 27.9 (12.7–62.2) | 32.8 (16.1–80.9) | 30.7 (14.7–81.7) |
|  | sEng(ng/ml) | 4.5 (3.5–5.6) | 5.5 (4.2–7.2) | 5.4 (4.2–7.1) |
|  | sFlt-1(pg/ml) | 2280 (1480–3580) | 2260 (1400–3650) | 2510 (1460–4310) |
|  | PlGF(pg/ml) | 456.3 (280.5–696.0) | 253.7 (122.4–396.0) | 153.6 (81.7–356.0) |
|  | sFlt-1/PlGF | 5.1 (2.9–9.3) | 8.3 (4.3–21.2) | 13.4 (4.7–42.5) |
|  | sEng(ng/ml) | 7.7 (5.6–11.0) | 17.4 (9.8–35.5) |  |
|  | sFlt-1(pg/ml) | 3760 (2520–5800) | 7905 (4750–13,620) |  |
|  | PlGF(pg/ml) | 513.8 (276.1–894.2) | 152.5 (79.0–249.7) |  |
|  | sFlt-1/PlGF | 6.9 (3.5–16.4) | 65.1 (20.5–158.3) |  |
| O'Gorman et al., 2017 | PAPP-A, PlGF |  |  |  |
| Clausen et al., 2002 | leptin (ng/ml) | 25 (16.0–35.0) | 19 (14.5–29.0) |  |
|  | TGF-beta1 (active form, ng/ml) | 3.2 (2.0–6.1) | 5.3 (3.8–7.1) |  |
|  | PAI-2 (ng/ml) | 78.8 (65.1–118.1) | 67.6 (61.6–79.6) |  |
| Honigberg et at., 2016 | PLGF (pg/ml) | 14.6 | 11.8 |  |
|  | sFLt-1(pg/ml) | 0.1 | 0.2 |  |
|  | PlGF(pg/ml) | 37.3 | 26.8 |  |
|  | sFlt-1(pg/ml) | -0.02 | 0.02 |  |
|  | PlGF(pg/ml) | -6.3 | -15.8 |  |
|  | sFlt-1(pg/ml) | 0.3 | 1.1 |  |
| Coolman et al., 2012 | sFlt-1 (ng/ml) | 5.14 (2.75–9.90) | 4.94 (2.45-9.82) |  |
|  | PlGF (pg/ml) | 43.5 (21.9–120) | 36.3 (17.4-111.6) |  |
|  | PAI-2 (ng/ml) | 39.8 (23.7–63.5) | 39.8 (23.7–65.6) |  |
|  | sFlt-1/PlGF | 0.00012 (0.00004–0.00028) |  |  |
|  | sFlt-1 (ng/ml) | 5.03 (2.31–11.3) |  |  |
|  | PlGF (pg/ml) | 203 (111–415) | 149 (64.8–341) |  |
|  | sFlt-1/PlGF | 0.000025 (0.000009–0.000063) | 0.000031 (0.000011–0.00013) |  |
| Schneuer et al., 2014 | Ang-1 (ng/ml) | 19.6 (13.8, 26.4) | 20.8 (15.4, 28.3) | 18.1 (16.1, 24.8) |
|  | Ang-2 (ng/ml) | 16.6 (11.0, 24.4) | 14.7 (9.2, 20.2) | 14.6 (7.8, 17.8) |
| Honarjoo et al., 2019 | beta-hCG (MoM) | 1.51 (SD=1.15) | 1.14 (SD=0.745) |  |
|  | PAPP-A (MoM) | 1 (SD=0.658) | 1.09 (0.585) |  |
| Mañé et al., 2019 | HbA1c (% +/- SD) |  |  |  |
| De Jonge et al., 2011 | CRP |  |  |  |
| Vieira et al., 2017 | adiponectin (ng/ml) | 4548 (3,467–5,809) (normal BMI), 3782 (2,892–4,965) (obese) |  |  |
|  | HDL cholesterol (MoM) | 1.03(0.89–1.18) (normal BMI), 0.92 (0.76–1.06) (obese) |  |  |
|  | ANP (ng/mL) | 0.5(0.2–0.9) (normal BMI), 0.4(0.2–0.8) (obese) |  |  |
|  | BNP (MoM) | 1.02 (0.73–1.46) (normal BMI), 0.94 (0.63–1.34) (obese) |  |  |
|  | Cystatin (ng/mL) | 1.813 (1.454–2.233) (normal BMI), 2.167(1.693–2.806) (obese) |  |  |
|  | sEng (ng/mL) | 16.9(13.3–21.8) (normal BMI), 12.9 (9.8–16.9) (obese) |  |  |
|  | PlGF (MoM) | 1.01(0.56–1.72) (normal BMI), 1.00 (0.55–1.70) (obese) |  |  |
| Herraiz et al., 2018 | sFlt-1 (pg/ml) | 1341 (median IQR= 880) | 2196, median IQR 1594 (intermediate), 1832 median IQR 562 (late) | 8627 (median IQR 6262) |
|  | PIGF (pg/ml) | 378.1 (median IQR 264.5) | 96.2, median IQR 121.6 (intermediate), 262.5, median IQR 357.2 (late) | 38.9, median IQR 41.2 |
|  | sFlt-1/PlGF | 3.7 (median IQR 3) | 22.4, median IQR 30.2 (intermediate), 6.1, median IQR 10.5 (late) | 239.8, median IQR 264.1 |
| Asvold et al., 2014 | hCG (IU/L) | 112 (77-157) | 113 (72-145) (mild PE) 93 (61-125) (severe PE) |  |
| Yung et al., 2019 | DAPK-1 |  |  |  |
| Boutin et al., 2017 | PAPP-A (MoM) |  | 0.82 (0.59-1.24) | 0.75 (0.54-1.02) |
| Kenny et al., 2014 | Angiogenin (mg/ml) | 6.9 (5.1–9.5) | 7.6 (5.6–11.0) |  |
|  | BNP (ng/ml) | 989 (686,1412) | 884 (631,1330) |  |
|  | CRP (ng/ml) | 9.9(5.3–18.5) | 12.6 (7.7–23.2) |  |
|  | cystatin (mg/ml) | 1.9 (1.5–2.4) | 2.0 (1.6–2.5) |  |
|  | elafin (ng/ml) | 124 (91-166) | 138 (97–178) |  |
|  | ICAM-1 (ng/ml) | 637 (513-799) | 672 (553–845) |  |
|  | IL-1Ra (ng/ml) | 10 (7-15) | 11 (8-17) |  |
|  | leptin (ng/ml) | 9.3 (5.5–15.5) | 11.3 (5.5-16.8) |  |
|  | leptin receptor (ng/ml) | 146 (109–182) | 132 (92-168) |  |
|  | PlGF (ng/ml) | 977 (545–1649) | 642 (347–1252) |  |
|  | TIMP-1 (ng/ml) | 91 (73–119) | 101 (74–131) |  |
| Schneuer et al., 2013 | PIGF (pg/ml) | 24.1 (18.3, 31.7) | 20.7 (17.2, 32.6) |  |
|  | sFlt-1 (pg/ml) | 286.8 (167.1, 472.1) | 268.1 (164.8, 390.5) |  |
|  | PAPP-A (pg/ml) | 1.71 (1.06, 2.79) | 1.34 (0.76, 2.4) |  |
| Rezk et al., 2018 | platelet volume (fL) | 7.79 | 7.82 |  |
|  | serum uric acid (mg/dl) | 4.2 | 4.3 |  |
|  | platelet volume (fL) | 8.51 | 9.89 |  |
|  | serum uric acid (mg/dl) | 5.62 | 7.66 |  |
| Sharma et al., 2018 | PAPP-A (mIU/ml) | 5.53± 3.03 | 3.99± 2.96 |  |
|  | hCG (ng/ml) | 49.78± 35.38 | 48.45± 32.70 |  |
|  | TNF-alpha (pg/ml) | 64.97± 17.48 | 80.4± 13.05 |  |
|  | INF-gamma (pg/ml) | 85.38± 22.85 | 94.11± 16.92 |  |
| Boutin et al., 2018 | PlGF, PAPP-A |  |  |  |
| Chaemsaithong et al., 2019 | PIGF |  |  |  |
| Boutin et al., 2020 | PAPP-A (mU/L) | 3742 (2355-5653) |  | 2235 (1856-3233) |
|  | PlGF (pg/mL) | 35.5 (27.3-46.3) |  | 21.6 (15.0-31.0) |
|  | sFlt-1 (pg/ml) | 1021.8 (771.0-1370.7) |  | 851.6 (657.9-1095.1) |
|  | AFP (ng/mL) | 15.3 (11.0-20.3) |  | 16.8 (11.1-23.4) |
|  | beta-hCG (ng/mL) | 31.5 (21.0-48.9) |  | 34.5 (25.9-52.1) |
| Sonek et al., 2018 | PIGF (IQR) | 1.01(0.81-1.27) | Late onset: 1.07 (0.84-1.28) | 0.68 (0.38-1.17) |
|  | PAPP-A (IQR) | 1.00 (0.69-1.50) | late onset: 0.97 (0.57-1.47) | 0.62 (0.50-0.86) |
|  | AFP (IQR) | 0.99 (0.74-1.33) | late onset: 0.96 (0.65-1.36) | 1.39 (1.01-1.49) |
| Chen et al., 2021 | LDH (U/L) | 150.03 ± 21.11 | 159.90 ± 31.13 |  |
|  | AST/ALT ratio | 1.19 ± 0.42 | 1.10 ± 0.42 |  |
|  | GGT (U/L) | 14.80 ± 8.63 | 23.71 ± 9.79 |  |
|  | ALP (U/L) | 49.02 ± 12.29 | 64.68 ± 30.19 |  |
|  | Uric acid (micromol/L) | 239.96 ± 49.34 | 273.81 ± 53.54 |  |
|  | eGFR (mL/min/1.73 m2) |  |  |  |
| Barjaktarovic et al., 2019 | hCG (IU/L) | 35 522.0 (6074.9–99 890.3) |  |  |
|  | sFlt-1/PlGF | 117.0 (19.5–432.6) (<18 GW) and 24.4 (5.3–106.8) (18-25 GW) - data refer to the whole population (no differentiation between PE and healthy pregnancies) | | |
| Stratieva et al., 2010 | PAPP-A (mU/L), median (range) | 2.82 (0.20-24.20) | 2.63 (0.32-9.88) late PE | 2.44 (0.11-8.69) |
| Wu et al., 2017 | SEPP1 polymorphisms |  |  |  |
| Tasleem et al., 2016 | leptin | 63.64 +/- 33.58 | 64.38 +/- 12.77 |  |
| Gonen et al., 2008 | PP13 (pg/ml) | 115 (95% CI 109–124) | 30.5 (95% CI 19–39) |  |
|  |  | 124 (95% CI 112–132) | 212.5 (95% CI 173–265) |  |
|  |  | 220.5 (95% CI 200–240) | 394 (95% CI 344–730) |  |
| Kusanovic et al., 2009 | sVEGFR-1 (pg/mL) | 1725 (48.6–13575.1) | 1426.3 (597.9–4546.5) |  |
|  | PlGF (pg/mL) | 33.8 (0.0–451.9) | 23.5 (0.0–77.1) |  |
|  | sEng (ng/mL) | 7.1 (3.3–26.9) | 7.4 (4.1–13.3) |  |
|  | sVEGFR-1 (pg/mL) | 1612 (245–10595.5) | 1637.4 (325.1–17768.9) |  |
|  | PlGF (pg/mL) | 329.8 (22.3–2894.4) | 213.9 (0.0–969.6) |  |
|  | sEng (ng/mL) | 5.9 (2.4–29.6) | 6.9 (3–47) |  |
| Hu et al., 2021 | PlGF (MoM) | 0.99 (0.73-1.32) | 0.83 (0.53-1.09) term PE | 0.91 (0.63-1.18) |
|  | PAPP-A (MoM) | 1.01 (0.69-1.44) | 0.86 (0.60-1.26) term PE | 0.70 (0.45-1.09) |
| Chaiyasit et al., 2022 | PlGF (pg/ml) | 37.32 (27.54-50.06) | 26.88 (18.89-237.61) |  |
|  | sFlt-1 (pg/ml) | 1563 (1176-2120) | 1480 (1095-2136) |  |
| Massé et al., 1993 | Hematocrit (%) | 33.6 +/- 2.3 | 34.0 +/- 2.2 |  |
|  | Mean corpuscular volume | 89.0 +/-3.5 | 87.6 +/-3.5 |  |
|  | Red blood cell distribution width (%) | 13.3 +/- 0.6 | 13.3 +/- 0.6 |  |
|  | Platelet count | 257 +/- 56 | 274 +/- 59 |  |
|  | Mean platelet volume | 9.2 +/- 1.0 | 9.0 +/- 1.0 |  |
|  | Antithrombin III | 1.05 +/- 0.10 | 1.07 +/- 0.08 |  |
|  | Haptoglobin (mg/dl) | 139 +/- 52 | 157 +/- 58 |  |
|  | Iron (ug/dl) | 106 +/- 33 | 105 +/- 32 |  |
|  | Transferrin (mg/dl) | 306 +/- 58 | 315 +/- 53 |  |
|  | Ferritin (ng/ml) | 60 +/- 47 | 69 +/- 52 |  |
|  | Total proteins (gmldl) | 6.60 +/- 0.34 | 6.64 +/- 0.38 |  |
|  | Albumin (gm/dl) | 3.82 +/- 0.21 | 3.79 +/- 0.19 |  |
|  | Calcium (mg/dl) | 8.55 +/- 0.36 | 8.59 +/- 0.30 |  |
|  | Magnesium (mg/dl) | 2.11 +/- 0.29 | 2.10 +/- 0.27 |  |
|  | Sodium (mEq/L) | 136.0 +/- 2.2 | 135.9 +/- 2.1 |  |
|  | Potassium (mEqlL) | 3.98 +/- 0.28 | 3.98 +/- 0.31 |  |
|  | Urea nitrogen (mg/dl) | 9.0 +/- 2.0 | 8.9 +/- 2.2 |  |
|  | Creatinine (mg/dl) | 0.74 +/- 0.08 | 0.75 +/- 0.08 |  |
|  | Uric acid (mg/dl) | 3.02 +/- 0.57 | 3.15 +/- 0.60 |  |
|  | Free estriol (ng/ml) | 2.59 +/ 1.60 | 2.49 +/- 1.45 |  |
|  | Progesterone (ng/ml) | 45.2 +/- 14.7 | 48.6 +/- 16.2 |  |
|  | Estriol/progesterone ratio | 0.063 +/- 0.064 | 0.058 +/- 0.052 |  |
| Boyle et al., 2016 | 25-hydroxyvitamin D (nmol/l) | 72.9 (SD=27) all participants (healthy + PE) | 68.1 (SD=27.8) | 70.6 (SD=29.0) |
| Mañé et al., 2017 | HbA1c |  |  |  |
| Panaitescu et al., 2018 | PlGF (MoM) | 1.019 (0.559–1.832) | 0.334 (0.198–0.556) |  |
|  | sFlt-1 (MoM) | 0.960 (0.701–1.370) | 2.147 (1.311–3.375) |  |
| Yue et al., 2020 | AFP (ng/mL) | 44.1 | 43.2 |  |
|  | hCG (mIU/L) | 57614 | 63863 |  |
|  | uE3 (ng/mL) | 1.05 | 0.97 |  |
|  | Inhibin A (pg/mL) | 220 | 265 |  |
| Ghosh et al., 2013 | PIGF (pg/ml) |  |  |  |
| Hirashima et al., 2018 | sLIGHT |  |  |  |
| Gybel-Brask et al., 2014 | YKL-40 (ug/ml) | 52 (20–155), 55 (20–145), 54 (20–152), 50 (20–180) | 67 (25–102), 75 (21–118), 83 (25–133), 70 (20–108) |  |
| Stonek et al., 2007 |  |  |  |  |
| Hanchard et al., 2020 | PAPP-A (MoM) | 1.46 (1.41-1.52) | 1.27 (1.10-1.47) |  |
|  | PlGF (MoM) | 1.09 (1.06-1.12) | 0.95 (0.84-1.06) |  |
|  | beta-hCG (MoM) | 1.14 (1.09-1.18) | 1.27 (1.01-1.53) |  |
|  | AFP (MOM) | 1.23 (1.19-1.26) | 1.21 (1.03-1.39) |  |
| Kumar et al., 2017 | PIGF (pg/ml) | 37.31 ± 13.28, median=34.2 | 30.42 ± 10.22, median=34.2 |  |
|  | PAPP-A (mIU/mL) | 5.57 ± 2.89, median=4.9 | 4.20 ± 2.95, median=4.9 |  |
|  | free beta-hCG (ng/ml) | 53.89 ± 38.04, median=43.9 | 48.36 ± 31.35, median=43.9, |  |
| Farzaneh et al., 2019 | AFP (MOM) | 2.17 +/- 0.78 | 2.65 +/- 0.89 |  |
|  | beta-HCG (MOM) | 2.27 +/- 0.90 | 2.68 +/- 1.10 |  |
|  | Inhibin A (MoM) | 1.67 +/- 0.59 | 1.98 +/- 0.81 |  |
|  | UE3 (MoM) | 1.08 +/- 0.43 | 0.88 +/- 0.50 |  |
| Kiely et al., 2016 | Serum 25(OH)D |  |  |  |

**Supplementary Table S5. Pregnancy-associated plasma protein A (PAPP-A).**

| **UNIT OF MEASUREMENT** | **STUDY** | **VALUE** | | | **OR, PLR, NLR, PPV, NPV** | **SENSITIVITY, SPECIFICITY** | **TRIMESTER (GW)** | **NUMBER OF PARTICIPANTS** | | |
| --- | --- | --- | --- | --- | --- | --- | --- | --- | --- | --- |
|  |  | **no condition** | **PE** | |  |  |  | **no PE** | **PE** | |
|  |  |  | ***any GW*** | ***⩽34 weeks (early onset PE)*** |  |  |  |  | ***any GW*** | ***⩽34 weeks (early onset PE)*** |
| MoM | Sonek et al. (2018) | 1.00 (0.69-1.50) | late onset: 0.97 (0.57-1.47) | 0.62 (0.50-0.86) | n/a | n/a | 1st (11-13 GW) | 1022 | 46 | 13 |
|  | Boutin et al. (2017) |  | 0.82 (0.59-1.24) | 0.75 (0.54-1.02) | n/a | n/a | 1st (10-13 GW) | 4240 | 232 | 30 |
|  | Hu et al., 2021 | 1.01 (0.69-1.44) | 0.86 (0.60-1.26) term PE | 0.70 (0.45-1.09) | n/a | n/a | 1st (11-13 GW) | 8333 | term PE: 195 | 117 |
|  | Hanchard et al., 2020 | 1.46 (1.41-1.52) | 1.27 (1.10-1.47) |  | n/a | n/a | 1st-2nd (10-14 GW) | 1086 | 55 (HDP) |  |
|  | Honarjoo et al. (2019) | 1 (SD=0.658) | 1.09 (0.585) |  | n/a | n/a | 1st (11-13 GW) | 4272 | 333 |  |
| pg/ml | Schneuer et al. (2013) | 1.71 (1.06, 2.79) | 1.34 (0.76, 2.4) |  | PPV=4.4% (1.6, 9.4)  NPV=97.6% (96.9, 98.1%)  LR=1.77 | 8.8% (3.3, 18.2) | 1st-2nd (12-14 GW) | 2468 | 68 |  |
| mIU/m | Boutin et al., 2020 | 3.742 (2.355-5.653) |  | 2.253 (1.856-3.233) | n/a | n/a | 1st (11-13 GW) | 3764 | 225 | 29 |
|  | Sharma et al. (2018) | 5.53± 3.03 | 3.99± 2.96 |  | PPV=60, NPV=77.5 | 72.9, 65.9 | 1st (11-13 GW) | 1454 | 199 |  |
|  | Kumar et al. (2017) | 5.57 ± 2.89, median=4.9 | 4.20 ± 2.95, median=4.9 |  | n/a | n/a | 1st (11-13 GW) | 1206 | 208 (HDP) | 74 (HDP) |
|  | Stratieva et al. (2010) | 2.82 (0.20-24.20) | 2.63 (0.32-9.88) late onset PE | 2.44 (0.11-8.69) | n/a | n/a | 1st (11-13 GW) | 8061 | 165 | 37 |
| no value provided | O'Gorman et al. (2017) |  |  |  | n/a | n/a | 1st (11-13 GW) | 8297 | 239 | 76 |
|  | Boutin et al. (2018) |  |  |  | n/a | n/a | 1st (11-13 GW) | 4420 | 232 | 30 |

**Supplementary Table S6. Soluble Fms-Like Tyrosine Kinase-1 (sFlt-1).**

| **UNIT OF MEASUREMENT** | **STUDY** | **VALUE** | | | **OR, PLR, NLR, PPV, NPV** | **SENSITIVITY, SPECIFICITY** | **TRIMESTER (GW)** | **NUMBER OF PARTICIPANTS** | | |
| --- | --- | --- | --- | --- | --- | --- | --- | --- | --- | --- |
|  |  | **no condition** | **PE** | |  |  |  | **no PE** | **PE** | |
|  |  |  | ***any GW*** | ***⩽34 weeks (early onset PE)*** |  |  |  |  | ***any GW*** | ***⩽34 weeks (early onset PE)*** |
| pg/ml | Widmer et al. (2015) | 2230 (1490–3340) | 1890 (1210–2840) | 2030 (1300–2930) | PLR, NLR, ORs, sensitivity and specificity provided by cut-off | | 1st-2nd (<20 GW) | 4929 | 198 | 47 |
|  |  | 2280 (1480–3580) | 2260 (1400–3650) | 2510 (1460–4310) |  |  | 2nd (23-27 GW) |  |  |  |
|  |  | 3760 (2520–5800) | 7905 (4750–13,620) |  |  |  | 3rd (32-35 GW) |  |  |  |
|  | Honigberg et al. (2016) | 0.1 | 0.2 |  | PPV=9.2%  NPV=92.9% | 53.5%, 53.4% | 1st-2nd (10-18 GW) | 2153 | 184 | 18 |
|  |  | -0.02 | 0.02 |  | PPV=9.7%  NPV=93.5% | 54.7%, 56.1% | 2nd (18-26 GW) |  |  |  |
|  |  | 0.3 | 1.1 |  | PPV=16.1%  NPV=96.8% | 70.5%, 70.5% | 2nd-3rd (26-35 GW) |  |  |  |
|  | Herraiz et al. (2018) | 1341 (median IQR= 880) | 2196, median IQR 1594 (intermediate), 1832 median IQR 562 (late) | 8627 (median IQR 6262) | Not provided | | 2nd-3rd (24-28 GW) | 5365 | 236 | 14 |
|  | Boutin et al., 2020 | 1021.8 (771.0-1370.7) |  | 851.6 (657.9-1095.1) | Not provided | | 1st (11-13 GW) | 3764 | 225 | 29 |
|  | Chaiyasit et al., 2022 | 1563 (1176-2120) | 1480 (1095-2136) |  | n/a | n/a | 1st (11-13 GW) | 7736 | 141 |  |
|  | Schneuer et al. (2013) | 286.8 (167.1, 472.1) | 268.1 (164.8, 390.5) |  | PPV=3.0% (0.8, 7.6), NPV=97.5% (96.8, 98.0), LR=1.18 | 5.9% (1.6, 14.4) | 1st-2nd (12-14 GW) | 2468 | 68 |  |
| ng/ml | Coolman et al. (2012) | 5.14 (2.75–9.90) | 4.94 (2.45-9.82) |  | Not provided | | 1st-2nd (<18 GW) | 7327 | 167 | 23 |
|  |  | 5.03 (2.31–11.3) | 5.20 (2.37–12.3) |  |  |  | 2nd (18-25 GW) |  |  |  |
| MoM | Panaitescu et al. (2018) | 0.960 (0.701–1.370) | 2.147 (1.311–3.375) |  | n/a | n/a | 3rd (35-37 GW) | 13078 | 272 |  |

**Supplementary Table S7. Beta human chorionic gonadotropin (hCG).**

| **UNIT OF MEASUREMENT** | **STUDY** | **VALUE** | | | **OR, PLR, NLR, PPV, NPV** | **SENSITIVITY, SPECIFICITY** | **TRIMESTER** | **NUMBER OF PARTICIPANTS** | | |
| --- | --- | --- | --- | --- | --- | --- | --- | --- | --- | --- |
|  |  | **no condition** | **PE** | |  |  |  | **no PE** | **PE** | |
|  |  |  | ***any GW*** | ***⩽34 weeks (early onset PE)*** |  |  |  |  | ***any GW*** | ***⩽34 weeks (early onset PE)*** |
| IU/L | Asvold et al. (2014) | 112 (77-157) | 113 (72-145) (mild PE) 93 (61-125) (severe PE) |  | n/a | n/a | 1st (12 days after embryo transfer) | 2300 | 105 (57 mild PE + 48 severe PE) |  |
|  | Barjaktarovic et al. (2019) | 35 522.0 (6074.9–99 890.3) |  |  | n/a | n/a | 1st-2nd (<18 GW) | 7303 | PE 165, hypertension 286 |  |
| ng/ml | Sharma et al. (2018) | 49.78± 35.38 | 48.45± 32.70 |  | n/a | n/a | 1st (11-13 GW) | 1801 | 45 |  |
|  | Boutin et al., 2020 | 31.5 (21.0-48.9) |  | 34.5 (25.9-52.1) | n/a | n/a | 1st (11-13 GW) | 3764 | 225 | 29 |
|  | Kumar et al. (2017) | 53.89 ± 38.04, median=43.9 | 48.36 ± 31.35, median=43.9, p=206 |  | n/a | n/a | 1st (11-13 GW) | 1206 | 208 (HDP) | 74 (HDP) |
| MoM | Farzanehet et al. (2019) | 2.27 +/- 0.90 | 2.68 +/- 1.10 |  | PPV=17.62, NPV=93.89 | 47.02%, 78.75% | 2nd (15-18 GW) | 1562 | 151 |  |
|  | Yue et al., 2020 | 1.11 ± 0.55 | 1.31 ± 0.72 |  | n/a | n/a | 2nd (14-20 GW) | 8333 | 560 |  |
|  | Hanchard et al., 2020 | 1.14 (1.09-1.18) | 1.27 (1.01-1.53) |  | n/a | n/a | 1st-2nd (10-14 GW) | 1086 | 55 (HDP) |  |
|  | Honarjoo et al. (2019) | 1.51 (SD=1.15) | 1.14 (SD=0.745) |  | n/a | n/a | 1st (11-13 GW) | 4272 | 333 |  |

**Supplementary Table S8. sFlt-1/PlGF.**

| **STUDY** | **VALUE** | | | **OR, PLR, NLR, PPV, NPV** | **SENSITIVITY, SPECIFICITY** | **TRIMESTER (GW)** | **NUMBER OF PARTICIPANTS** | | |
| --- | --- | --- | --- | --- | --- | --- | --- | --- | --- |
|  | **no condition** | **PE** | |  |  |  | **no PE** | **PE** | |
|  |  | ***any GW*** | ***⩽34 weeks (early onset PE)*** |  |  |  |  | ***any GW*** | ***⩽34 weeks (early onset PE)*** |
| Widmer et al. (2015) | 27.9 (12.7–62.2) | 32.8 (16.1–80.9) | 30.7 (14.7–81.7) | PLR, NLR, ORs, sensitivity and specificity provided by cut-off | | 1st-2nd (<20 GW) | 4929 | 198 | 47 |
|  | 5.1 (2.9–9.3) | 8.3 (4.3–21.2) | 13.4 (4.7–42.5) |  |  | 2nd (23-27 GW) |  |  |  |
|  | 6.9 (3.5–16.4) | 65.1 (20.5–158.3) |  |  |  | 3rd (32-35 GW) |  |  |  |
| Coolman et al. (2012) | 0.00012 (0.00004–0.00028) | 0.00012 (0.000033–0.00041) |  | Not provided | | 1st-2nd (<18 GW) | 7327 | 167 | 23 |
|  | 0.000025 (0.000009–0.000063) | 0.000031 (0.000011–0.00013) |  |  |  | 2nd (18-25 GW) |  |  |  |
| Herraiz et al. (2018) | 3.7 (median IQR 3) | 22.4, median IQR 30.2 (intermediate), 6.1, median IQR 10.5 (late) | 239.8, median IQR 264.1 | Not provided | | 2nd-3rd (24-28 GW) | 5365 | 236 | 14 |
| Barjaktarovic et al. (2019) | 117.0 (19.5–432.6) (<18 GW) and 24.4 (5.3–106.8) (18-25 GW) - data refer to the whole population (no differentiation between PE and healthy pregnancies) | | | n/a | n/a | 1st-2nd (<18 GW) | 7303 | 165 |  |
|  |  |  |  | n/a | n/a | 2nd (18-25 GW) |  |  |  |

**Supplementary Table S9. Leptin.**

| **UNIT OF MEASUREMENT** | **STUDY** | **VALUE** | | | **OR, PLR, NLR, PPV, NPV** | **SENSITIVITY, SPECIFICITY** | **TRIMESTER** | **NUMBER OF PARTICIPANTS** | | |
| --- | --- | --- | --- | --- | --- | --- | --- | --- | --- | --- |
|  |  | **no condition** | **PE** | |  |  |  | **no PE** | **PE** | |
|  |  |  | ***any GW*** | ***⩽34 weeks (early onset PE)*** |  |  |  |  | ***any GW*** | ***⩽34 weeks (early onset PE)*** |
| ng/ml | Clausen et al. (2002) | 25 (16.0–35.0) | 19 (14.5–29.0) |  | n/a | n/a | 2nd trimester (18 GW) | 2119 | 71 |  |
|  | Kenny et al. (2014) | 9.3 (5.5–15.5) | 11.3 (5.5-16.8) |  | n/a | n/a | 2nd (14-16 GW) | 5317 | 306 | 28 |
|  | Tasleem et al. (2016) | 63.64 +/- 33.58 | 64.38 +/- 12.77 |  | n/a | n/a | 2nd (16 GW) | 1087 | 134 |  |

**Supplementary Table S10. Soluble Endoglin (sEng).**

| **UNIT OF MEASUREMENT** | **STUDY** | **VALUE** | | | **OR, PLR, NLR, PPV, NPV** | **SENSITIVITY, SPECIFICITY** | **TRIMESTER** | **NUMBER OF PARTICIPANTS** | | |
| --- | --- | --- | --- | --- | --- | --- | --- | --- | --- | --- |
|  |  | **no condition** | **PE** | |  |  |  | **no PE** | **PE** | |
|  |  |  | ***any GW*** | ***⩽34 weeks (early onset PE)*** |  |  |  |  | ***any GW*** | ***⩽34 weeks (early onset PE)*** |
| pg/ml | Widmer et al. (2015) | 5.0 (3.9–6.4) | 5.5 (4.3–7.6) | 5.8 (4.3–8.4) | PLR, NLR, ORs, sensitivity and specificity provided by cut-off | | 1st-2nd (<20 GW) | 4929 | 198 | 47 |
|  |  | 4.5 (3.5–5.6) | 5.5 (4.2–7.2) | 5.4 (4.2–7.1) |  |  | 2nd (23-27 GW) |  |  |  |
|  |  | 7.7 (5.6–11.0) | 17.4 (9.8–35.5) |  |  |  | 3rd (32-35 GW) |  |  |  |
| ng/ml | Vieira et al. (2017) | 16.9(13.3–21.8) (normal BMI), 12.9 (9.8–16.9) (obese) | 18.8(14.4–25.5) (normal BMI), 13.6 (9.3–19.5) (obese) | | OR=1.65 (1.07, 2.55) in normal BMI  OR= 1.26 (0.79–1.99) in obese women | n/a | 1st-2nd (10-16 GW) | 3767 | 182 |  |
|  | Kusanovic et al. (2009) | 7.1 (3.3–26.9) | 7.4 (4.1–13.3) |  | PPV=7.1%, NPV=97.1%, PLR=1.9 (1.4-2.6), NLR= 0.8 (0.6-0.9) | 40.3%, 79% | 1st-2nd (6-15 GW) | 1560 | 62 |  |
|  |  | 5.9 (2.4–29.6) | 6.9 (3–47) |  | PPV=8%, NPV=97.8%, PLR=2.2 (1.7-2.6), NLR= 0.6 (0.4-0.7) | 58.1, 73.3 | 2nd (20-25 GW) |  |  |  |

**Supplementary Table S11. Alpha fetoprotein (AFP).**

| **UNIT OF MEASUREMENT** | **STUDY** | **VALUE** | | | **OR, PLR, NLR, PPV, NPV** | **SENSITIVITY, SPECIFICITY** | **TRIMESTER** | **NUMBER OF PARTICIPANTS** | | |
| --- | --- | --- | --- | --- | --- | --- | --- | --- | --- | --- |
|  |  | **no condition** | **PE** | |  |  |  | **no PE** | **PE** | |
|  |  |  | ***any GW*** | ***⩽34 weeks (early onset PE)*** |  |  |  |  | ***any GW*** | ***⩽34 weeks (early onset PE)*** |
| MoM | Farzaneh et al., 2019 | 2.17 +/- 0.78 | 2.56 +/- 0.89 |  | PPV=19.60, NPV=95.63 | 64.90%, 74.26% | 2nd (15-18 GW) | 1562 | 151 |  |
|  | Hanchard et al., 2020 | 1.23 (1.19-1.26) | 1.21 (1.03-1.39) |  | n/a | n/a | 1st-2nd (10-14 GW) | 1086 | 55 (HDP) |  |
| Median (IQR) | Sonek et al., 2018 | 0.99 (0.74-1.33) | late onset: 0.96 (0.65-1.36) | 1.39 (1.01-1.49) | n/a | n/a | 1st (11-13 GW) | 1022 | late onset: 33 | 13 |
| ng/mL | Boutin et al., 2020 | 15.3 (11.0-20.3) |  | 16.8 (11.1-23.4) | n/a | n/a | 1st (11-13 GW) | 3764 | 225 | 29 |
|  | Yue et al., 2020 | 44.1 | 43.2 |  | n/a | n/a | 2nd (14-20 GW) | 8333 | 560 |  |

**Supplementary Table S12. Uric acid (UA).**

| **UNIT OF MEASUREMENT** | **STUDY** | **VALUE** | | **OR, PLR, NLR, PPV, NPV** | **SENSITIVITY, SPECIFICITY** | **TRIMESTER** | **NUMBER OF PARTICIPANTS** | |
| --- | --- | --- | --- | --- | --- | --- | --- | --- |
|  |  | **no condition** | **PE (any GW)** |  |  |  | **no PE** | **PE (any GW)** |
| mg/dL | Massé et al., 1993 | 3.02 +/-0.57 | 3.15 +/- 60 | n/a | n/a | 2nd (15-24) | 1116 | 109 |
|  | Rezk et al., 2018 | 4.2 +/- 1.3 | 4.3 +/- 1.2 | n/a | n/a | 1st (10-12 GW) | 9236 | 286 |
|  |  | 5.62 +/- 0.72 | 9.89 +/- 0.8 | PPV=83.3%, NPV=83.3%, | 95.2%, 55.6% | 2nd (18-20 GW) |  |  |
| micromol/L | Chen et al., 2021 | 239.96 ± 49.34 | 273.81 ± 53.54 | n/a | n/a | 1st-2nd (<18 GW) | 981 | 31 |

**Supplementary Table S13. Unconjugated Estriol (UE3).**

| **UNIT OF MEASUREMENT** | **STUDY** | **VALUE** | | **OR, PLR, NLR, PPV, NPV** | **SENSITIVITY, SPECIFICITY** | **TRIMESTER** | **NUMBER OF PARTICIPANTS** | |
| --- | --- | --- | --- | --- | --- | --- | --- | --- |
|  |  | **no condition** | **PE** |  |  |  | **no PE** | **PE** |
|  |  |  | ***any GW*** |  |  |  |  | ***any GW*** |
| MoM | Farzanehet et al. (2019) | 1.08 ± 0.43 |  | PPV=14.88% , NPV=93.57% | 47.68%, 73.62% | 2nd (15-18 GW) | 1562 | 151 |
| ng/ml | Yue et al. (2020) | 1.11 ± 0.55 | 0.97 ± 0.35 | n/a | n/a | 2nd (14-20 GW) | 8333 | 560 |
|  | Massé et al. (1993) | 2.59 ±1.6 | 2.49 ± 1.45 | n/a | n/a | 2nd (15-24 GW) | 1116 | 109 |

**Supplementary Table S14. Other biomarkers identified.**

| **BIOMARKER** | **STUDY** |  |
| --- | --- | --- |
|  |  |  |
|  |  |  |
| PAI-2 | Clausen et al., 2002 |  |
|  | Coolman et al., 2012 |  |
| HbA1c | Mañé et al., 2019 |  |
|  | Mañé et al., 2017 |  |
| CRP | De Jonge et al., 2011 |  |
|  | Kenny  et al., 2014 |  |
| BNP | Kenny et al., 2014 |  |
|  | Vieira et al., 2017 |  |
| cystatin | Vieira et al., 2017 |  |
|  | Kenny et al., 2014 |  |
| platelet | Rezk et al., 2018 |  |
|  | Massé et al., 1993 |  |
| Cystatin C | Kenny et al., 2014 |  |
|  | Vieira et al., 2017 |  |
| Inhibin A | Farzaneh et al., 2019 |  |
|  | Yue et al., 2020 |  |
| TNF-α | Sharma et al., 2018 |  |
| leptin receptor | Kenny et al., 2014 |  |
| TGF-β1 | Clausen et al., 2002 |  |
| Ang-1 | Schneuer et al., 2014 |  |
| Ang-2 | Schneuer et al., 2014 |  |
| adiponectin | Vieira et al., 2017 |  |
| HDL cholesterol | Vieira et al., 2017 |  |
| ANP | Vieira et al., 2017 |  |
| angiogenin | Kenny et al., 2014 |  |
| elafin | Kenny et al., 2014 |  |
| ICAM-1 | Kenny et al., 2014 |  |
| IL-1Ra | Kenny et al., 2014 |  |
| TIMP-1 | Kenny et al., 2014 |  |
| INF-γ | Sharma et al., 2018 |  |
| SEPP1 polymorphism | Wu et al., 2017 |  |
| PP13 | Gonen at al., 2008 |  |
| sVEGFR | Kusanovic et al., 2009 |  |
| Hematocrit | Massé et al., 1993 |  |
| MCV | Massé et al., 1993 |  |
| Red blood cell distribution width (%) | Massé et al., 1993 |  |
| Mean platelet volume | Massé et al., 1993 |  |
| Antithrombin III | Massé et al., 1993 |  |
| Haptoglobin | Massé et al., 1993 |  |
| Iron | Massé et al., 1993 |  |
| Transferrin | Massé et al., 1993 |  |
| Ferritin | Massé et al., 1993 |  |
| Total proteins | Massé et al., 1993 |  |
| Albumin | Massé et al., 1993 |  |
| Calcium | Massé et al., 1993 |  |
| Magnesium | Massé et al., 1993 |  |
| Sodium | Massé et al., 1993 |  |
| Potassium | Massé et al., 1993 |  |
| Urea nitrogen | Massé et al., 1993 |  |
| Creatinine | Massé et al., 1993 |  |
| Progesterone | Massé et al., 1993 |  |
| Estriol/progesterone ratio | Massé et al., 1993 |  |
| sLIGHT | Hirashima et al., 2018 |  |
| YJL-40 | Gybel-Brask et al., 2014 |  |
| LDH | Chen et al., 2021 |  |
| AST/ALT ratio | Chen et al., 2021 |  |
| GGT | Chen et al., 2021 |  |
| ALP | Chen et al., 2021 |  |
| eGFR | Chen et al., 2021 |  |
| DAPK-1 | Yung et al. 2019 |  |
